# Potency Analysis of Semi-Synthetic Cannabinoids in Vaping Oils Using Liquid Chromatography Diode Array Detector with Electrospray Ionization Time-of-Flight Mass Spectrometry for Confirmation of Analyte Identity

**DOI:** 10.3390/molecules30122597

**Published:** 2025-06-15

**Authors:** Shaozhong Zhang, Md Mahmud Alam, Brent D. Chandler, Jocelyn P. Lanorio, Caitlin Deskins, Liguo Song

**Affiliations:** 1 Department of Chemistry, Western Illinois University, Macomb, IL 61455, USA; s-zhang@wiu.edu (S.Z.); mm-alam2@wiu.edu (M.M.A.); 2 Department of Chemistry, Illinois College, Jacksonville, IL 62650, USA; brent.chandler@ic.edu (B.D.C.); jocelyn.lanorio@ic.edu (J.P.L.); 3 Department of Chemistry, Quincy University, Quincy, IL 62301, USA; deskica@quincy.edu

**Keywords:** potency analysis, semi-synthetic cannabinoids, liquid chromatography diode array detector, electrospray ionization time-of-flight mass spectrometry

## Abstract

Since the 2018 Farm Bill legalized hemp, semi-synthetic cannabinoids, typically derived from hemp-extracted CBD, have been marketed as offering a “legal high”, raising concerns about consumer safety, labeling, and regulation. Consequently, the potency analysis of these compounds has become increasingly important. To address this need, an LC-DAD method was developed for the quantification of seventeen cannabinoids, selected based on the synthetic pathways of semi-synthetic cannabinoids. These included naturally occurring compounds, semi-synthetic derivatives, and byproducts (CBC, CBD, CBDV, CBG, CBN, CBN-O-acetate, CBT, 9(R)-HHC, 9(S)-HHC, 9(R)-HHC-O-acetate, 9(S)-HHC-O-acetate, Δ^8^-THC, Δ^9^-THC, Δ^9,11^-THC, Δ^8^-THC-O-acetate, Δ^9^-THC-O-acetate, and Δ^9^-THCV), using abnormal CBD as an internal standard. The method was validated according to ISO 17025 guidelines, demonstrating a linear calibration range from 0.1 to 50 µg/mL. The method was further applied to the potency analysis of one Δ^8^-THC, two THC-O-acetate, two HHC, and one HHC-O-acetate vaping oil sample. Using an innovative method to recover the contents of vaping cartridges, cannabinoids were extracted using methanol, diluted to a concentration of 50 µg/mL, and analyzed using the validated LC-DAD method, which provided a quantifiable range of 0.1 to 100% (*w*/*w*). Method specificity was evaluated using ESI/TOFMS and showed minimal interference, despite the presence of other isomers of the semi-synthetic cannabinoids in the samples.

## 1. Introduction

*Cannabis sativa* L. has long been a subject of legal and scientific controversy due to its unique ability to produce cannabinoids, a class of naturally occurring compounds with significant pharmacological effects [1,2,3,4]. Among the most prominent of these are Δ^9^-tetrahydrocannabinol (Δ^9^-THC) and cannabidiol (CBD). Δ^9^-THC is the primary psychotropic component of the plant and is responsible for the “high” associated with marijuana use. Because of its intoxicating properties, which can manifest at concentrations as low as 1% [1], marijuana was classified as a Schedule I controlled substance under the Controlled Substances Act (CSA) of 1970. In contrast, CBD has emerged in recent years as a non-intoxicating cannabinoid with a promising therapeutic profile [5,6]. This change in scientific understanding and public perception played a key role in the passage of the 2018 Farm Bill, which reclassified hemp as a legal agricultural commodity. Under the bill, hemp is defined as “the plant *Cannabis sativa* L. and any part of that plant, including the seeds thereof and all derivatives, extracts, cannabinoids, isomers, acids, salts, and salts of isomers, whether growing or not, with a delta-9 tetrahydrocannabinol concentration of not more than 0.3 percent on a dry weight basis”.

The 2018 Farm Bill represented a major turning point in cannabis regulation, opening the door for the legal cultivation, scientific investigation, and commercial distribution of hemp-derived products across the United States. However, the broad language of the 2018 Farm Bill inadvertently created a regulatory gray area, which some manufacturers have exploited by producing and marketing semi-synthetic cannabinoids, such as Δ^8^-THC, THC-O-acetate, hexahydrocannabinol (HHC), and HHC-O-acetate [7,8,9,10,11,12,13]. These compounds are typically synthesized from CBD extracted from legal hemp and are often promoted as offering a “legal high”, raising significant concerns regarding consumer safety, product labeling, and regulatory oversight. In response, the potency analysis of these semi-synthetic cannabinoids has become increasingly important.

**Figure 1:**
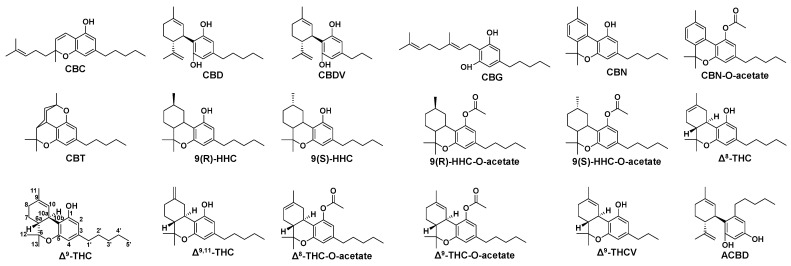
Chemical structure of eighteen cannabinoids.

In *Cannabis sativa* L. plants, cannabinoids are initially biosynthesized in their acidic forms (Supplementary Figure S1) [2,3,4]. Cannabigerolic acid (CBGA) is first formed from olivetolic acid via geranyltransferase. From CBGA, cannabidiolic acid (CBDA), Δ^9^-tetrahydrocannabinolic acid (Δ^9^-THCA), and cannabichromenic acid (CBCA) are produced through the action of their respective synthase enzymes. When exposed to heat, either in the plant or post-harvest, these acidic cannabinoids decarboxylate into their neutral forms: cannabigerol (CBG), CBD, Δ^9^-THC, and cannabichromene (CBC). Additional neutral cannabinoids like Δ^8^-THC, cannabinol (CBN), and cannabicitran (CBT) arise through heat-induced reactions such as isomerization, decomposition, and oxidation. The plant also produces minor homologs of CBD and Δ^9^-THC, such as cannabidivarin (CBDV) and Δ^9^-tetrahydrocannabivarin (Δ^9^-THCV), which differ by having a three-carbon side chain instead of the typical five-carbon chain (Figure 1).

As illustrated in Supplementary Figure S2, CBD can undergo intramolecular cyclization under acidic conditions, where one of its phenolic hydroxyl groups adds with its isopropenyl group, forming Δ^9^-THC. Once Δ^9^-THC is formed, it can be protonated at the carbon-10 position, generating a tertiary carbocation at the carbon-9 position that facilitates isomerization primarily into Δ^8^-THC, along with smaller amounts of Δ^9,11^-THC (also known as exo-THC). Due to the low specificity of these acid-catalyzed isomerization reactions, commercial Δ^8^-THC products often contain a mixture of Δ^8^-THC, Δ^9^-THC, and Δ^9,11^-THC. To circumvent legal restrictions on Δ^9^-THC, chemical modifications are increasingly used to create analogs that may fall outside the regulatory definitions of THC. One common approach is the hydrogenation of Δ^8^-THC products, which produces two stereoisomers, 9(R)- and 9(S)-HHC, as shown in Supplementary Figure S3. Another strategy involves acetylation, resulting in Δ^8^- and Δ^9^-THC-O-acetate, as well as 9(R)- and 9(S)-HHC-O-acetate, as illustrated in Supplementary Figure S4 and Figure S5, respectively.

The objective of this study was to develop a method for the potency analysis of semi-synthetic cannabinoids. Based on the synthetic pathways outlined in Supplementary Figures S1–S5 and a preliminary study, seventeen cannabinoids were identified for quantification (Figure 1). These include all the naturally occurring neutral cannabinoids covered by the AOAC International’s Standard Method Performance Requirements (SMPRs) for potency analysis in cannabis concentrates [14], namely CBC, CBD, CBDV, CBG, CBN, and Δ^9^-THCV, except for Δ^8^- and Δ^9^-THC, which were treated as semi-synthetic in this context due to their derivation from CBD. CBT was also included, as it frequently exceeded the SMPR limit of quantification (LOQ), i.e., 0.3% (*w*/*w*) [14,15,16]. These naturally occurring neutral cannabinoids may appear as impurities in the CBD used for synthesizing semi-synthetic compounds. They may also be intentionally added to products to enhance their diversity. Additionally, some, such as CBT, could be synthetic byproducts. Most importantly, this study specifically focused on a range of semi-synthetic compounds and byproducts, including Δ^8^-, Δ^9^-, and Δ^9,11^-THC; 9(R)- and 9(S)-HHC; Δ^8^- and Δ^9^-THC-O-acetate; and 9(R)- and 9(S)-HHC-O-acetate. CBN-O-acetate was included in the list following a preliminary study that revealed its high contents in a couple of samples. Additionally, abnormal CBD (ACBD), a cannabinoid not naturally found in hemp and not structurally related to THC, was used as an internal standard.

Liquid chromatography (LC) is widely recognized as the preferred method for the potency analysis of cannabinoids in hemp-based products, as gas chromatography (GC) can cause the decarboxylation of acidic cannabinoids due to its high-temperature conditions [17,18,19,20,21,22,23,24,25,26]. For LC detection, diode array detection (DAD) is generally favored over electrospray ionization tandem mass spectrometry (ESI/MS/MS) because it is more accessible to crime laboratories, commercial suppliers, and farmers. However, LC-DAD depends on achieving the baseline separation of all cannabinoids and any unknown compounds present above the required LOQ [14]. In addition, isocratic separation is preferred over gradient separation in LC-DAD, as changes in the mobile phase can lead to baseline drift when detecting neutral cannabinoids at 230 nm or shorter wavelengths, which increases the LOQ [15].

Since the potency analysis of semi-synthetic cannabinoids does not involve acidic cannabinoids, both the GC and LC techniques can be used. However, a validated method for a comprehensive potency analysis of these compounds is still lacking [27,28,29,30,31,32,33,34,35,36]. To address this gap, the present study aimed to develop an LC-DAD method capable of quantifying widely used semi-synthetic cannabinoids, along with their synthetic byproducts and naturally occurring neutral cannabinoids present in the samples.

## 2. Results and Discussion

### 2.1. Separation Optimization

A recent study by us investigated the separation of ten naturally occurring neutral cannabinoids, including CBC, CBD, CBDV, CBG, cannabicyclol (CBL), CBN, CBT, Δ^8^-THC, Δ^9^-THC, and Δ^9^-THCV, using four different columns with identical dimensions (150 mm × 2.1 mm, 2.7 µm) but differing in stationary phases: -OSi(CH_3_)_2_C_18_H_37_ (Poroshell 120 EC-C18), -OSi(*i*Bu)_2_C_18_H_37_ (Raptor ARC-18), -OSi(CH_3_)_2_C_3_H_6_O(CO)NHC_12_H_25_ (Cortecs Shield RP-18), and -OSi(CH_3_)_2_C_3_H_6_NH(CO)C_15_H_31_ (Ascentis Express RP-Amide) [37]. The mobile phase consisted of 0.02% (*v*/*v*) formic acid in water as the A solvent and acetonitrile as the B solvent, with varying contents of B solvent, and was delivered at a flow rate of 0.3 mL/min.

The baseline separation of all ten cannabinoids was successfully achieved with the Poroshell 120 EC-C18 column at 75.0 and 70.0% (*v*/*v*) acetonitrile, in 16.5 and 25.0 min, respectively. In contrast, the Raptor ARC-18 column did not yield baseline separation. Both the Cortecs Shield RP-18 and Ascentis Express RP-Amide columns achieved baseline separation at 65.0% (*v*/*v*) acetonitrile, with retention times of 14.5 and 26 min, respectively.

In this study, a Poroshell 120 EC-C18 column was employed to separate the eighteen cannabinoids (Figure 1) using 75.0% (*v*/*v*) acetonitrile in the mobile phase. Supplementary Figure S6A presents the LC-UV (ultraviolet) chromatogram at 208 nm, while Supplementary Figure S6B displays the corresponding LC-ESI/TOFMS extracted ion chromatograms (EICs) based on the [M+H]^+^ ions of the eighteen cannabinoids within a ±20 ppm mass window. The key critical pairs identified for separation included CBG/CBD, Δ^9,11^-/Δ^9^-THC, Δ^9^-/Δ^8^-THC, 9(S)-/9(R)-HHC, and 9(R)-HHC/CBC, with respective resolution values of 1.56, 1.41, 1.38, 1.94, and 1.04.

**Figure 2:**
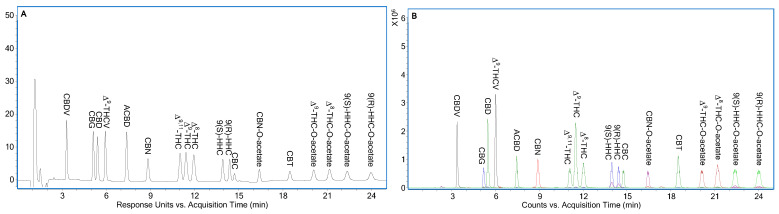
Optimized separation of eighteen cannabinoids in Figure 1. (**A**) LC-UV chromatogram at 208 nm. (**B**) Corresponding LC-ESI/TOFMS EICs of eighteen cannabinoids in Figure 2A using their [M+H]^+^ ions with ±20 ppm. A solvent was 0.02% (*v*/*v*) formic acid. B solvent was acetonitrile. Mobile phase contained 70.0% (*v*/*v*) B. Mobile phase flow rate was set at 0.3 mL/min from 0 to 13.5 min and 0.6 mL/min from 13.51 to 25 min. Eighteen cannabinoids were at 1 µg/mL individual concentration.

Improved separation was achieved using 70.0% (*v*/*v*) acetonitrile, with a flow rate of 0.3 mL/min from 0 to 13.5 min and 0.6 mL/min from 13.51 to 25 min, as shown in Figure 2. Under these conditions, the resolution values for the same critical pairs changed to 1.63, 1.38, 1.78, 2.11, and 1.48, respectively. Although the Δ^9,11^-/Δ^9^-THC pair exhibited a resolution less than 1.5, this was deemed acceptable given that both compounds were byproducts of semi-synthetic cannabinoids and were expected to be present only in trace contents. In contrast, the baseline separation of semi-synthetic compounds from adjacent peaks was considered essential to enable accurate quantification.

Both the Cortecs Shield RP-18 and Ascentis Express RP-Amide columns were also evaluated for the separation of the eighteen cannabinoids (Figure 1) using 65.0% (*v*/*v*) acetonitrile in the mobile phase. However, the resolution of the Δ^9,11^-/Δ^9^-THC pair was inadequate and deemed unacceptable.

### 2.2. Method Validation

Supplementary Figure S7 presents the UV absorption spectra of eighteen cannabinoids extracted from Figure 2A. Most cannabinoids, including CBDV, CBG, CBD, Δ^9^-THCV, ACBD, Δ^9,11^-THC, Δ^9^-THC, Δ^8^-THC, 9(S)-HHC, 9(R)-HHC, and CBT, exhibited similar UV spectra, characterized by two peaks around 208 nm and 228 nm. CBN showed distinct peaks at approximately 221 nm and 282 nm, while CBC displayed peaks at about 228 nm and 282 nm. CBN-O-acetate had three peaks at approximately 215 nm, 273 nm, and 308 nm. Compared to Δ^9^-THC, Δ^8^-THC, 9(S)-HHC, and 9(R)-HHC, Δ^9^-THC-O-acetate, Δ^8^-THC-O-acetate, 9(R)-HHC-O-acetate, and 9(S)-HHC-O-acetate exhibited similar dual peaks, though slightly blue-shifted by 2 to 4 nm. As a result, LC-UV data acquisition was conducted at 208, 221, 228, 273, and 282 nm. For quantification, the shorter-wavelength peak of each cannabinoid was generally selected for its higher signal-to-noise (S/N) ratio, except in the cases of 9(S)-HHC, 9(R)-HHC, and CBC at 228 nm, and CBN-O-acetate at 273 nm, where lower background interference near the LOQ was observed (see details below).

Supplementary Figure S8 shows the ESI/TOFMS spectra of the eighteen cannabinoids extracted from Figure 2A, including their *m/z* values and retention times. All cannabinoids predominantly produced [M+H]^+^ ions, which were subsequently used to confirm analyte identities during sample analysis.

Method validation was based on the LC separation shown in Figure 2 and was conducted three times per day across three separate days. Due to the simplicity of the procedure and its good performance, external standard calibration was selected over internal standard calibration. Linear calibration curves were established over the concentration range of 0.1 to 50 µg/mL by plotting the peak area of each cannabinoid against its concentration, applying a 1/x² weighting factor. The lowest coefficient (R²) among all calibration curves was 0.9887, as shown in Supplementary Table S1.

To assess accuracy and precision, quality control (QC) samples at concentrations of 0.1, 1, and 50 µg/mL were analyzed. The results met ISO 17025 requirements: for low-concentration QCs, both intraday and interday accuracy and precision fell within 80–120% and below 20%, respectively, while for medium- and high-concentration QCs, values remained within 85–115% and below 15%, respectively.

Supplementary Table S2 summarizes the accuracy of the QC samples. At 0.1 µg/mL, intraday accuracy ranged from 94.2% to 114.6%, and interday accuracy ranged from 96.8% to 111.3%. At 1 µg/mL, intraday accuracy was between 96.2% and 104.2%, while interday accuracy ranged from 97.0% to 100.7%. At 50 µg/mL, intraday accuracy fell between 101.1% and 110.7%, and interday values ranged from 102.8% to 109.5%.

Supplementary Table S3 shows the precision results. At 0.1 µg/mL, the relative standard deviation (RSD) was less than 11.3% for intraday and less than 3.7% for interday. At 1 µg/mL, the RSD was below 4.7% intraday and below 3.4% interday. At 50 µg/mL, the RSD was under 3.7% for intraday and under 2.4% for interday.

Supplementary Table S4 presents uncertainty estimates for cannabinoid quantification, based on precision data from both intraday and interday replicate measurements of QC samples (Supplementary Table S3). The combined standard uncertainty was calculated by pooling the standard deviations of intraday replicates and integrating them with the interday standard deviation.

**Figure 3:**
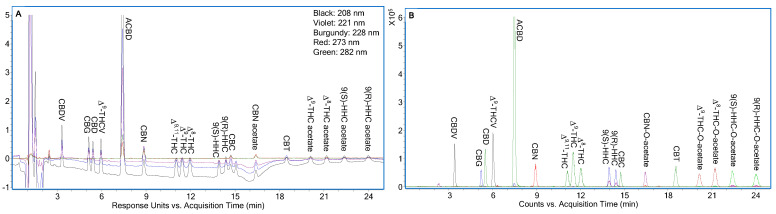
(**A**) LC-UV chromatograms of the eighteen cannabinoids at LOQ level, i.e., 0.1 µg/mL, except for ACBD at 1 µg/mL under optimized conditions as shown in Figure 2; (**B**) Corresponding LC-ESI/TOFMS EICs of the eighteen cannabinoids in Figure 3A using their [M+H]^+^ ions with ±20 ppm.

Figure 3A shows LC-UV chromatograms of individual cannabinoids at 0.1 µg/mL and ACBD at 1 µg/mL, recorded at 208, 221, 228, 273, and 282 nm. A change in the mobile phase flow rate from 0.3 to 0.6 mL/min at 13.51 min caused a temporary baseline disturbance, which stabilized after approximately 5 min. During this period, detection at longer wavelengths offered better signal clarity due to reduced baseline interference. For instance, 9(S)-HHC, 9(R)-HHC, and CBC showed improved detection at 228 nm over 208 nm, and CBN-O-acetate maintained a stable baseline at 273 nm. Based on Figure 3A and a linear calibration range of 0.1 to 50 µg/mL, it was determined that the LOQ of the method was to be reported as 0.1 µg/mL for all the cannabinoids, although it could be lower for early eluting cannabinoids. Figure 3B displays the corresponding LC-ESI/TOFMS EICs of the cannabinoids shown in Figure 3A.

To track sample preparation recovery in real time, ACBD, a cannabinoid not naturally found in hemp and not structurally related to THC, was spiked into each sample at 1.5% (*w*/*w*). The average recovery of ACBD across triplicates was 98.2% for the Delta-8 THC Vape Cartridge—1 Gram—Sour Diesel from Serene Tree (Serene Tree D8), 99.1% for the HHC Vape Cartridge (Flavor: Cali Gold) from Binoid (Binoid HHC), 101.4% for the iDELTA Premium Diamond—HHC Cartridge Full Gram (Flavor: Northern Light) from iDELTA8 (iDELTA8 HHC), and 102.4% for the HHC-O Vape Cartridge (Flavor: Forbidden Fruit) from Binoid (Binoid HHCO). The corresponding RSD values were 3.6%, 1.8%, 2.2%, and 1.2%, respectively. Recovery could not be calculated for the Delta 9o Vape Cartridge—Snowman—1 mL from 3Chi (3Chi D9O) and the iDELTA Premium Diamond—THCO Cartridge Full Gram (Flavor: Pineapple Express) from iDELTA8 (iDELTA8 D9O) due to the presence of an interfering peak (see details below).

### 2.3. Sample Analysis

Ciolino et al. [35] previously described a complex method for recovering vaping oils from vaping cartridges. In this study, a simpler and innovative approach was employed. Both ends of the cartridge were removed and discarded, and the cartridge was placed into approximately 20 mL of pre-weighed methanol in a 50 mL centrifuge tube. The combined weight of the cartridge and methanol was recorded. Ultrasonication effectively dissolved the vaping oil into the methanol. The methanol solution was then recovered, and the cartridge was rinsed with methanol, dried, and re-weighed to determine the amount of oil recovered and calculate its concentration.

Six vaping oil samples were analyzed in triplicate using the validated LC-DAD method. As summarized in Table 1, thirteen of the seventeen targeted cannabinoids were detected, with average contents ranging from 0.3% to 62.2% (*w*/*w*). The RSDs for triplicate measurements ranged from 0.3% to 10.2%, as shown in Table 2.

The Serene Tree D8 sample contained a high content of Δ^8^-THC as the targeted semi-synthetic cannabinoid, accompanied by a notable concentration of Δ^9^-THC (approximately one-tenth of the Δ^8^-THC level) and slightly lower concentration of Δ^9,11^-THC (around one-third of the Δ^9^-THC content) as the byproducts, suggesting a synthetic pathway consistent with Supplementary Figure S2. Trace levels of CBN and CBT were also detected, likely as impurities originating from the CBD used in the semi-synthesis of Δ^8^-THC.

The 3Chi D9O sample contained a high content of Δ^8^-THC-O-acetate as the targeted semi-synthetic cannabinoid, accompanied by a notable concentration of Δ^9^-THC-O-acetate (approximately one-fourth of the Δ^8^-THC-O-acetate level), suggesting a synthetic pathway consistent with Supplementary Figures S2 and S4 where Δ^8^-THC served as an intermediate and Δ^8^-THC-O-acetate was the final product. The relatively high content of CBN-O-acetate (approximately one-eighth of the Δ^8^-THC-O-acetate level) certainly originated from a high initial CBN content; however, it remained uncertain whether CBN was intentionally added or formed as a byproduct during the semi-synthesis of Δ^8^-THC. Similarly, the presence of trace contents of 9(R)-HHC was difficult to interpret.

The iDELT8 D9O sample contained a high content of Δ^9^-THC-O-acetate as the targeted semi-synthetic cannabinoid, along with low contents of Δ^8^-THC-O-acetate, Δ^8^-THC, and CBD, suggesting a synthetic route distinct from that of the 3Chi D9O sample. This composition indicated that Δ^9^-THC, rather than CBD, might be used as the starting material, which actually aligned with the product’s name. In contrast, the 3Chi D9O sample actually contained high content of Δ^8^-THC-O-acetate, which did not align with the product’s name.

The Binoid HHC sample contained high contents of both 9(S)-HHC and 9(R)-HHC, with 9(R)-HHC present at a relatively higher level. This composition suggested a synthetic pathway consistent with Supplementary Figures S2 and S3, in which Δ^8^-THC served as an intermediate and both HHC isomers were final products. Notably, the content of CBN exceeded that of 9(R)-HHC. Given that oxidation of Δ^8^-THC yields CBN while its reduction produces HHC, CBN could arise as a byproduct during semi-synthesis of Δ^8^-THC or be intentionally added. In consideration of its unusually high level, it was more likely added deliberately.

The iDELT8 HHC sample also contained high contents of both 9(S)-HHC and 9(R)-HHC, with 9(R)-HHC more abundant. It also included low concentrations of Δ^8^-THC and CBD, along with trace level of CBN. This composition suggested a synthetic pathway consistent with Supplementary Figures S2 and S3, where Δ^8^-THC served as an intermediate and both HHC isomers were final products.

The Binoid HHCO sample contained high contents of both 9(S)-HHC-O-acetate and 9(R)-HHC-O-acetate, with 9(R)-HHC-O-acetate being more abundant. It also exhibited high contents of CBN-O-acetate and Δ^8^-THC, along with low concentrations of 9(S)-HHC and 9(R)-HHC, with the latter again being more abundant. Additionally, low concentrations of Δ^9^-THC and CBN were detected, as well as trace level of Δ^9,11^-THC. This composition suggested a synthetic route consistent with Supplementary Figures S2, S3 and S5: Δ^8^-THC acting as an intermediate in the first to second step, both HHC isomers serving as intermediates in the second to third step, and 9(S)- and 9(R)-HHC-O-acetate as the final products. Considering the unusually high level of CBN-O-acetate, it was likely added deliberately in an early stage of synthesis. In contrast, the high content of Δ^8^-THC suggested it might be intentionally added to the final product.

### 2.4. Assessment of Specificity

**Figure 4:**
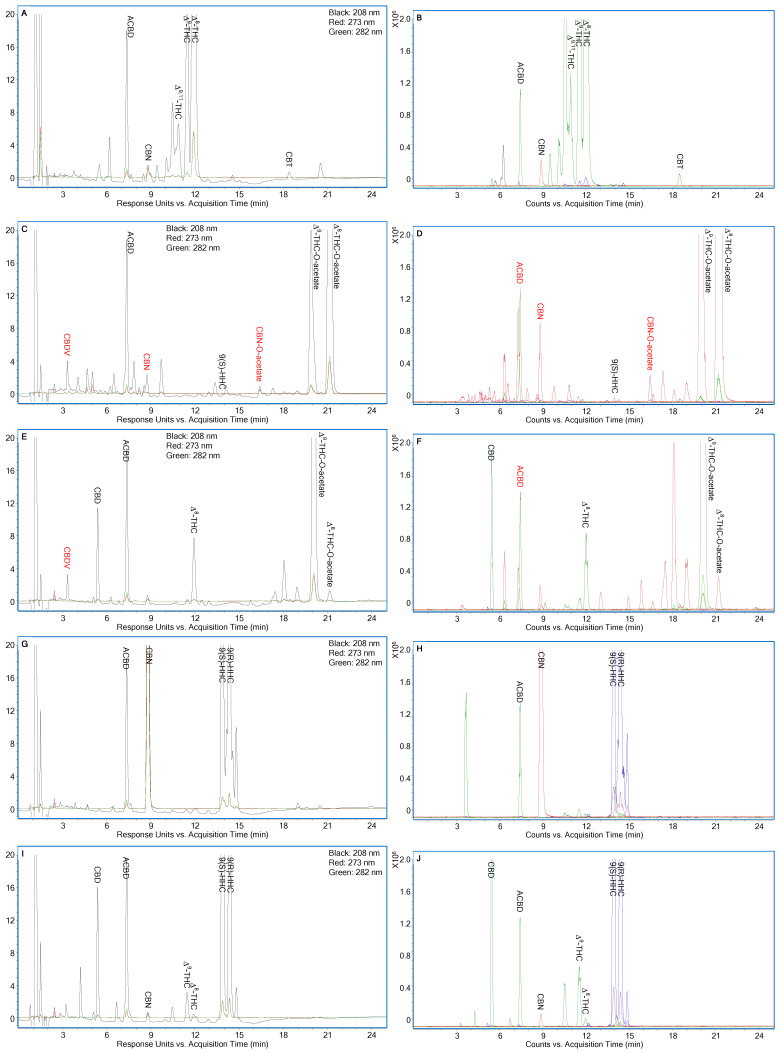

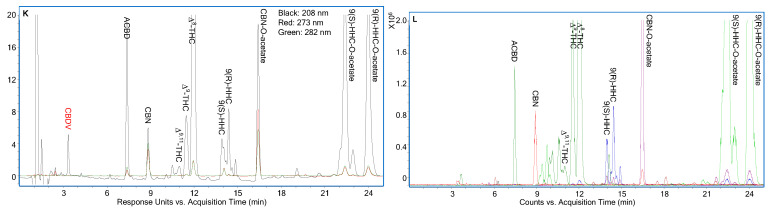
(**A**,**C**,**E**,**G**,**I**,**K**) LC-UV chromatograms of 50 µg/mL Serene Tree D8, 3Chi D9O, iDELT8 D9O, Binoid HHC, iDELT8 HHC, and Binoid HHCO, all spiked with 1.5% (*w*/*w*) ACBD, under optimized conditions, as shown in Figure 2, respectively. (**B**,**D**,**F**,**H**,**J**,**L**) Corresponding LC-ESI/TOFMS EICs of cannabinoids in (**A**,**C**,**E**,**G**,**I**,**K**) using their [M+H]^+^ ions with ±20 ppm.

Method specificity was assessed using ESI/TOFMS following UV detection. Figure 4A,C,E,G,I,K display the LC-UV chromatograms of 50 µg/mL Serene Tree D8, 3Chi D9O, iDELT8 D9O, Binoid HHC, iDELT8 HHC, and Binoid HHCO, each spiked with 1.5% (*w*/*w*) ACBD and analyzed under the optimized conditions outlined in Figure 2. The corresponding LC-ESI/TOFMS EICs for the neutral cannabinoids in these samples are shown in Figure 4B,D,F,H,J,L.

Although numerous unknown peaks were observed in the LC-UV chromatograms, most did not interfere with the quantification of the targeted cannabinoids. However, quantification could be less accurate in cases of inadequate resolution, as demonstrated in Figure 4A,K, where other THC isomers eluted earlier than Δ^9,11^-THC, resulting in overlapping peaks. While our previous studies detected other THC isomers beyond Δ^9,11^-, Δ^9^-, and Δ^8^-THC, this study was the first to detect isomers of 9(S)-/9(R)-HHC, Δ^8^-/Δ^9^-THC-O-acetate, and 9(S)-/9(R)-HHC-O-acetate. Despite their presence, these isomers are unlikely to significantly affect the quantification of the main semi-synthetic cannabinoids due to either sufficient chromatographic resolution or their relatively low concentrations. However, as intermediate products, the quantification of 9(S)- and 9(R)-HHC may be less accurate due to inadequate resolution from an overlapping peak during the analysis of Binoid HHCO (Figure 4K,L).

A few minor interferences could be avoided with careful interpretation. The LC-UV chromatograms in Figure 4C,E,K indicated the presence of CBDV; however, its identification was ruled out by the LC-ESI/TOFMS EICs in Figure 4D,F,L. Even in hemp plant materials, CBDV and Δ^9^-THCV are rarely detected at concentrations greater than 0.1% (*w*/*w*), even when taking into account their precursors, cannabidivarinic acid (CBDVA) and Δ^9^-tetrahydrocannabivarinic acid (Δ^9^-THCVA) [15,16]. In purified CBD starting materials for the semi-synthesis of cannabinoids, their content should be even lower unless intentionally added as naturally occurring cannabinoids to diversify the products. In such cases, their concentrations should be well above the LOQ.

The LC-UV chromatogram in Figure 4C did not identify CBN due to the absence of a peak at 282 nm, although a peak was observed at 208 nm, a finding further confirmed by the LC-ESI/TOFMS EICs in Figure 4D. In Figure 4C,D, the quantification of CBN-O-acetate at 273 nm was not affected by an adjacent peak detected at 208 nm. However, in Figure 4C,E, the coeluting peak with ACBD in the LC-UV chromatograms was not visible due to insufficient separation, which was identified as one of the isomers of Δ^8^-/Δ^9^-THC-O-acetate in the LC-ESI/TOFMS EICs shown in Figure 4D,F.

## 3. Materials and Methods

### 3.1. Chemicals and Reagents

LC-grade water, acetonitrile, methanol, and formic acid were obtained from Fisher Scientific (Pittsburgh, PA, USA). Certified reference materials (CRMs) of cannabinoid standards were obtained from Cayman Chemical (Ann Arbor, MI, USA). These included a Phytocannabinoid Neutrals Mixture 8 solution in acetonitrile, containing CBC, CBD, CBDV, CBG, CBN, Δ^8^-THC, Δ^9^-THC, and Δ^9^-THCV, each at a concentration of 1 mg/mL. Additionally, separate solutions of Δ^9,11^-THC and CBT in methanol were acquired, both at 1 mg/mL. Further CRMs in acetonitrile at 1 mg/mL included 9(R)-HHC, 9(S)-HHC, 9(R)-HHC-O-acetate, 9(S)-HHC-O-acetate, Δ^8^-THC-O-acetate, and Δ^9^-THC-O-acetate. Abnormal CBD (ACBD), a cannabinoid not naturally produced by hemp and not structurally related to THC, was obtained at a concentration of 25 mg/mL in methyl acetate and spiked into each sample to monitor recovery in real-time during sample preparation.

### 3.2. Calibration Solutions and Quality Control Samples

A mixture solution containing seventeen cannabinoids was initially prepared in methanol at an individual concentration of 100 µg/mL. This solution was then serially diluted with methanol to produce eight additional solutions with individual cannabinoid concentrations of 50, 25, 10, 5, 2, 1, 0.4, and 0.2 µg/mL. Separately, an ACBD solution was prepared in methanol at a concentration of 2 µg/mL. Each cannabinoid mixture solution was then combined with the 2 µg/mL ACBD solution at a 1:1 (*v*/*v*) ratio, resulting in nine calibration solutions containing 1 µg/mL ACBD and individual cannabinoid concentrations of 50, 25, 12.5, 5, 2.5, 1, 0.5, 0.2, and 0.1 µg/mL. Quality control (QC) samples containing 1 µg/mL ACBD and 50, 1, and 0.1 µg/mL of each cannabinoid were prepared in the same manner.

### 3.3. Samples

Six vaping cartridges were purchased, including Delta-8 THC Vape Cartridge-1 Gram-Sour Diesel from Serene Tree (Vista, CA, USA), Delta 9o Vape Cartridge–Snowman–1 mL from 3Chi (Carmel, IN, USA), iDELTΔ Premium Diamond–THCO Cartridge Full Gram (Flavor: Pineapple Express) from iDELT8 (Phoenix, AZ, USA), HHC VAPE CARTRIDGE (Flavor: Cali Gold) from Binoid (Northridge, CA, USA), iDELTΔ Premium Diamond–HHC Cartridge Full Gram (Flavor: Northern Light) from iDELT8, and HHC-O VAPE CARTRIDGE (Flavor: Forbidden Fruit) from Binoid.

### 3.4. Sample Preparation

To recover vaping oil from a vaping cartridge, its exterior was cleaned with methanol, and both end covers were removed and discarded. About 20 mL of methanol was added to a pre-weighed 50 mL centrifuge tube, and the weight of the methanol was recorded. The cartridge was then added to the tube, and the total weight was measured. The mixture was sonicated for several hours with a Branson Ultrasonic Cleaner (Brookfield, CT, USA) to extract the vaping oil. The resulting solution was transferred as completely as possible to another 50 mL centrifuge tube. The empty cartridge was rinsed with methanol, dried, and weighed to determine the amount of vaping oil recovered for concentration (*w*/*w*) calculation.

To obtain approximately 10 mg of vaping oil, the required volume of its methanol solution was calculated using the oil’s concentration (*w*/*w*) and the density of methanol (0.791 mg/µL).

To prepare 4 mL of an approximately 2.5 mg/mL vaping oil solution, the required volume of methanol was determined by subtracting the volume of the vaping oil solution from 2000 µL. This volume of methanol and 2000 µL of 75 µg/mL ACBD in methanol were added to a 15 mL centrifuge tube. The required volume of vaping oil solution was added, and its weight was recorded. The exact concentration of vaping oil in the final solution was calculated.

Cannabinoids were extracted by vortexing the solution for 15 s, followed by ultrasonication for 5 min. The mixture was then centrifuged at 13,000 rpm (15.8× *g*) for 10 min using an Eppendorf centrifuge (Hamburg, Germany). The supernatant was filtered through a 0.2 µm PTFE syringe filter (Foxx Life Science, Salem, NH, USA) and subsequently diluted 50-fold for analysis.

### 3.5. LC-DAD

LC-DAD analysis was performed using an Agilent 1260 Infinity II LC system (Agilent Technologies, Santa Clara, CA, USA) equipped with a solvent degasser, binary pump, temperature-controlled autosampler, column oven, and DAD. Chromatographic separation was achieved using an Agilent Poroshell 120 EC-C18 column (150 mm × 2.1 mm, 2.7 µm), preceded by a 0.2 µm ultra-high-pressure (UHP) pre-column filter (IDEX Health & Science, Oak Harbor, WA, USA). The column was housed in the oven at 30 °C. The A solvent was 0.02% (*v*/*v*) formic acid. The B solvent was acetonitrile. The mobile phase was 70% (*v*/*v*) B. The mobile phase flow rate was set at 0.3 mL/min from 0 to 13.5 min and 0.6 mL/min from 13.51 to 25 min. The autosampler was maintained at 8 °C, and the injection volume was 4 µL. UV detection was conducted at 208, 221, 228, 273, and 282 nm with a 4 nm bandwidth, using a reference wavelength of 360 nm with a 100 nm bandwidth. UV spectra were recorded over a range of 190.0 to 400.0 nm with a step size of 2.0 nm.

### 3.6. ESI/TOFMS

ESI/TOFMS was performed using an Agilent 6545 quadrupole time-of-flight (Q-TOF) mass spectrometer equipped with a Dual Agilent Jet Stream (AJS) ESI source. The ESI source operated in positive ion mode, generating predominant [M+H]^+^ ions. Optimized mass spectrometry parameters were as follows: mass range, 100–1000 *m*/*z*; acquisition rate, 5 spectra/s; drying gas temperature, 325 °C; drying gas flow rate, 10 L/min; nebulizer pressure, 20 psi; sheath gas temperature, 400 °C; sheath gas flow rate, 12 L/min; capillary voltage, 3000 V; nozzle voltage, 600 V; fragmentor voltage, 120 V; skimmer voltage, 45 V; Oct1 RF Vpp, 750 V. Reference mass ions used for continuous calibration were *m*/*z* 121.0509 and 922.0098.

## 4. Conclusions

An LC-DAD method was developed for quantifying seventeen cannabinoids, including CBDV, CBG, CBD, Δ^9^-THCV, CBN, Δ^9,11^-THC, Δ^9^-THC, Δ^8^-THC, 9(S)-HHC, 9(R)-HHC, CBC, CBN-O-acetate, CBT, Δ^9^-THC-O-acetate, Δ^8^-THC-O-acetate, 9(S)-HHC-O-acetate, and 9(R)-HHC-O-acetate, for the potency analysis of commonly available semi-synthetic cannabinoid products on the market. LC optimization led to the use of an Agilent Poroshell 120 EC-C18 column (150 mm × 2.1 mm, 2.7 µm), 0.02% (*v*/*v*) formic acid as the A solvent, acetonitrile as the B solvent, 70% (*v/v*) B as the mobile phase, and a flow rate of 0.3 mL/min from 0 to 13.5 min and 0.6 mL/min from 13.51 to 25 min. Method validation complied with ISO 17025 guidelines, exhibiting a linear calibration range of 0.1–50 µg/mL.

The validated method was applied to the potency analysis of one Δ^8^-THC, two THC-O-acetate, two HHC, and one HHC-O-acetate vaping oil sample. Cannabinoids were extracted using methanol, then diluted to 50 µg/mL to allow for quantification across a 0.1–100% (*w*/*w*) range. Thirteen of the seventeen targeted cannabinoids were detected, with average contents ranging from 0.3% to 62.2% (*w*/*w*) and RSDs between 0.3% and 10.2% for triplicate measurements. Extraction recovery was monitored in real time using spiked ACBD at 1.5% (*w*/*w*), a cannabinoid not naturally found in hemp but readily available and cost-effective, as an internal standard. Recoveries ranged from 98.2% to 102.4%, with RSDs of 1.2–3.6%. ESI/TOFMS was used to assess method specificity, confirming minimal interference, although other isomers of the semi-synthetic cannabinoids were observed in the samples.

## Figures and Tables

**Table 1:** Average content (% *w*/*w*) of cannabinoids in vaping cartridges (triplicates; cannabinoid-O-acetate abbreviated as x-O-, where x = any cannabinoid).

Sample	CBD	CBN	Δ^9,11^-THC	Δ^9^-THC	Δ^8^-THC	9(S)-HHC	9(R)-HHC
Serene Tree D8		0.6	2.2	5.9	60.2		
3Chi D9O							0.4
iDELT8 D9O	2.0				2.4		
Binoid HHC		30.6				17.4	25.1
iDELT8 HHC	2.8	0.4			0.3	30.4	34.3
Binoid HHCO		2.1	0.7	2.4	20.9	1.3	2.1
Sample		CBN-O-	CBT	Δ^9^-THC-O-	Δ^8^-THC-O-	9(S)-HHC-O-	9(R)-HHC-O-
Serene Tree D8			0.8				
3Chi D9O		7.8		16.0	62.2		
iDELT8 D9O				55.2	1.0		
Binoid HHC							
iDELT8 HHC							
Binoid HHCO		11.0				19.8	24.5

**Table 2:** RSD values (%) of cannabinoids in vaping cartridges (triplicates; cannabinoid-O-acetate abbreviated as x-O-, where x = any cannabinoid).

Sample	CBD	CBN	Δ^9,11^-THC	Δ^9^-THC	Δ^8^-THC	9(S)-HHC	9(R)-HHC
Serene Tree D8		4.9	5.3	5.5	5.4		
3Chi D9O							3.1
iDELT8 D9O	0.4				0.3		
Binoid HHC		3.4				2.8	3.2
iDELT8 HHC	3.1	0.3			1.9	1.8	2.1
Binoid HHCO		1.5	1.2	1.9	1.4	2.6	1.2
Sample		CBN-O-	CBT	Δ^9^-THC-O-	Δ^8^-THC-O-	9(S)-HHC-O-	9(R)-HHC-O-
Serene Tree D8			7.5				
3Chi D9O		10.2		3.3	3.5		
iDELT8 D9O				1.2	2.9		
Binoid HHC							
iDELT8 HHC							
Binoid HHCO		1.5				1.0	1.8

## Data Availability

The datasets generated during and/or analyzed during the current study are available from the corresponding author upon reasonable request.

## Supplementary Materials

The following supporting information can be downloaded at https://www.mdpi.com/article/10.3390/molecules30122597/s1. Supplementary Figure S1. Bio-synthetic pathways of natural cannabinoids. Supplementary Figure S2. Synthetic pathways of THC isomers: Δ^9^-/Δ^8^-/Δ^9,11^-THC. Supplementary Figure S3. Synthetic pathways of THC analogs: Δ^9^-/Δ^8^-THC-O-acetate. Supplementary Figure S4. Synthetic pathways of THC analogs: 9(S)/9(R)-HHC. Supplementary Figure S5. Synthetic pathways of THC analogs: 9(S)/9(R)-HHC-O-acetate. Supplementary Figure S6. The LC separation of the eighteen cannabinoids using an Agilent Poroshell 120 EC-C18 column (150 mm × 2.1 mm, 2.7 µm): (A) The LC-UV chromatogram at 208 nm. (B) The corresponding LC-ESI/TOFMS EICs of the eighteen cannabinoids in Figure S6A using their [M+H]^+^ ions with ±20 ppm. The A solvent was 0.02% (*v*/*v*) formic acid. The B solvent was acetonitrile. The mobile phase contained 75.0% (*v*/*v*) B. The flow rate was 0.3 mL/min. The eighteen cannabinoids were at 1 µg/mL individual concentration. Supplementary Figure S7. The UV absorption spectra of the eighteen cannabinoids that were extracted from Figure 2A. Supplementary Figure S8. The ESI/TOFMS spectra of the eighteen cannabinoids that were extracted from Figure 2A. Supplementary Table S1. Summary of calibration curves (cannabinoid-O-acetate abbreviated as x-O-, where x = any cannabinoid). Supplementary Table S2. The accuracy (%) of the QC samples: triplicate measurements for both intraday and interday. Supplementary Table S3. The precision of the QC samples: triplicate measurements for both intraday and interday. Supplementary Table S4. Uncertainty estimates for cannabinoid quantification.
