# Supplementary material for: Potency Analysis of Semi-Synthetic Cannabinoids in Vaping Oils Using Liquid Chromatography Diode Array Detector with Electrospray Ionization Time-of-Flight Mass Spectrometry for Confirmation of Analyte Identity
Source: Molecules. 2025 Jun 15;30(12):2597. doi: 10.3390/molecules30122597 (PMC12195824; doi:10.3390/molecules30122597)
Supplement: Supplementary file 1 [file molecules-30-02597-s001.zip › molecules-3680856-supplementary.pdf]

Supplementary Figure S1. Biosynthetic pathways of natural cannabinoids.

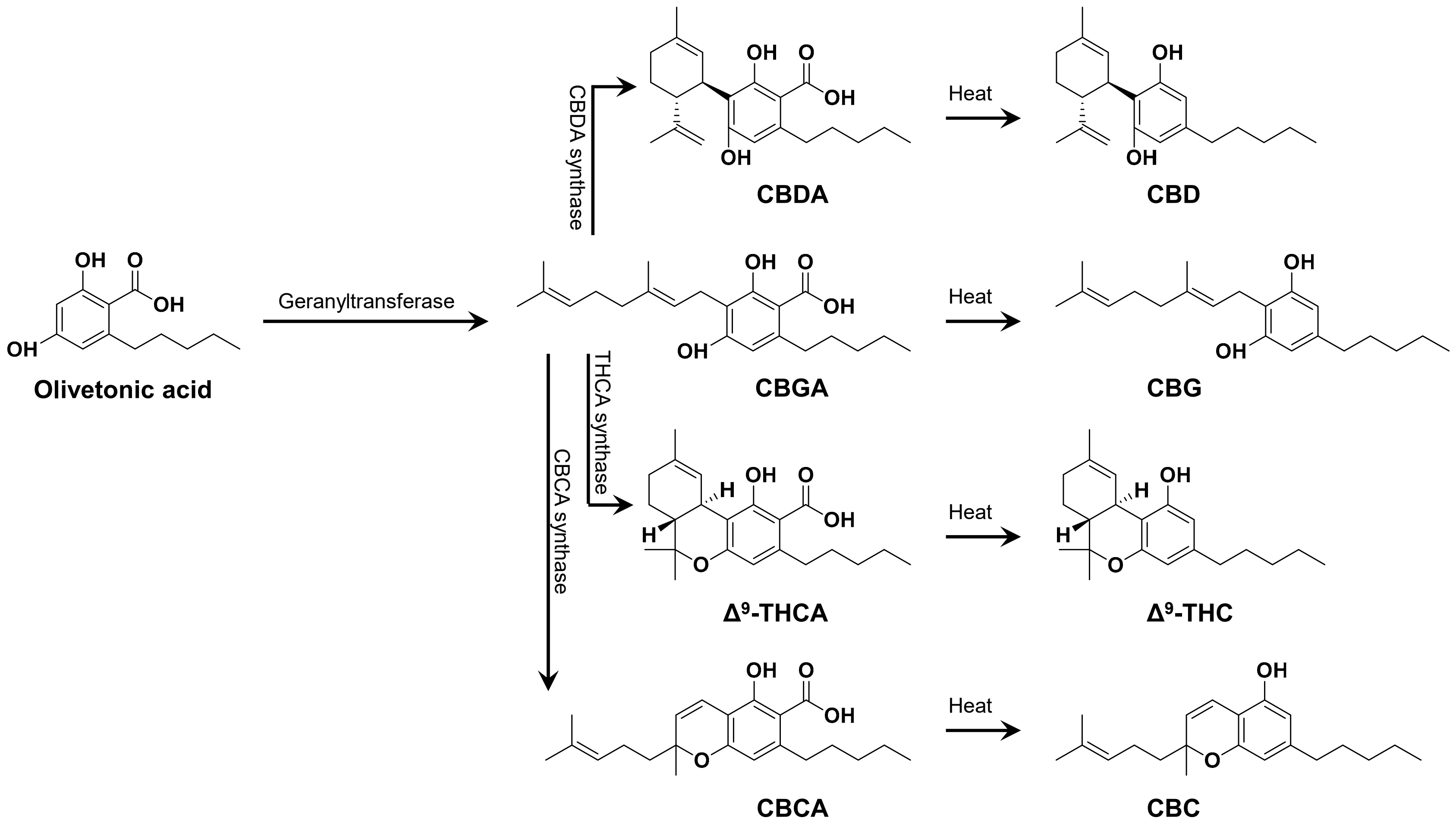

Supplementary Figure S2. Synthetic pathways of THC isomers: Δ^9-/Δ^8-/Δ^{9,11}-THC.

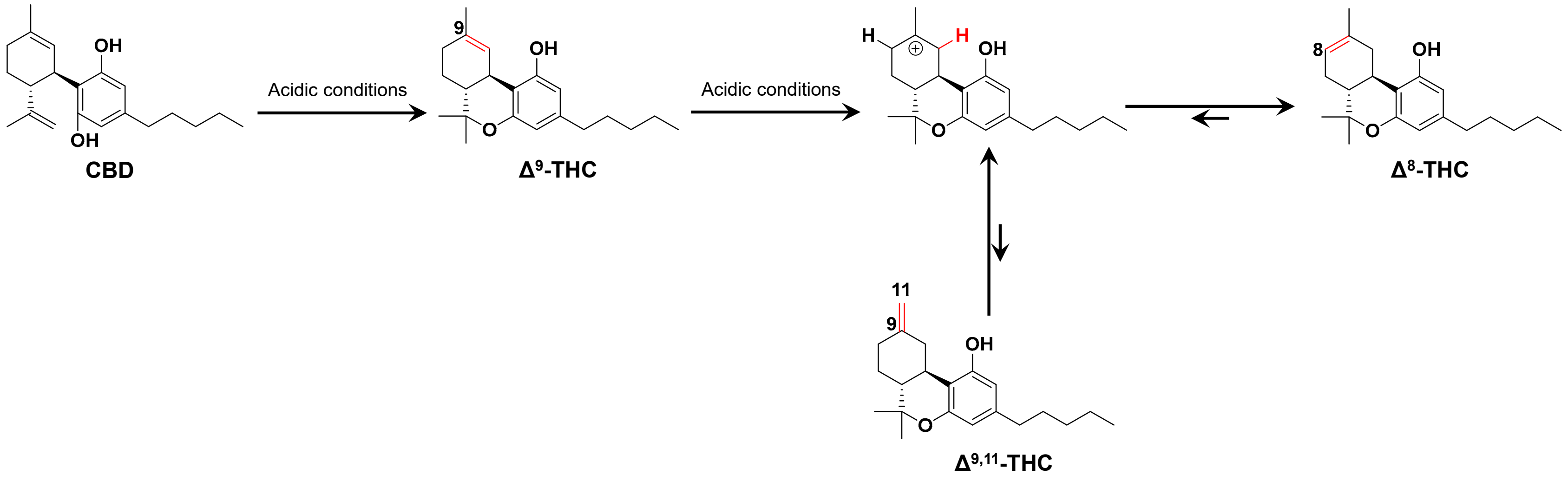

Supplementary Figure S3. Synthetic pathways of THC analogs: 9(S)/9(R)-HHC.

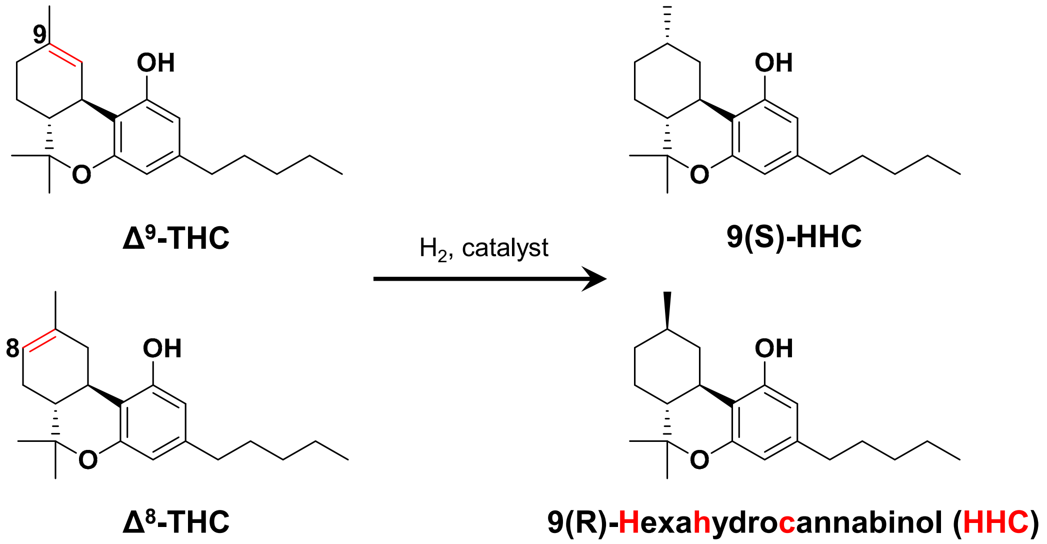

Supplementary Figure S4. Synthetic pathways of THC analogs: Δ^9-/Δ^8-THC-O-acetate.

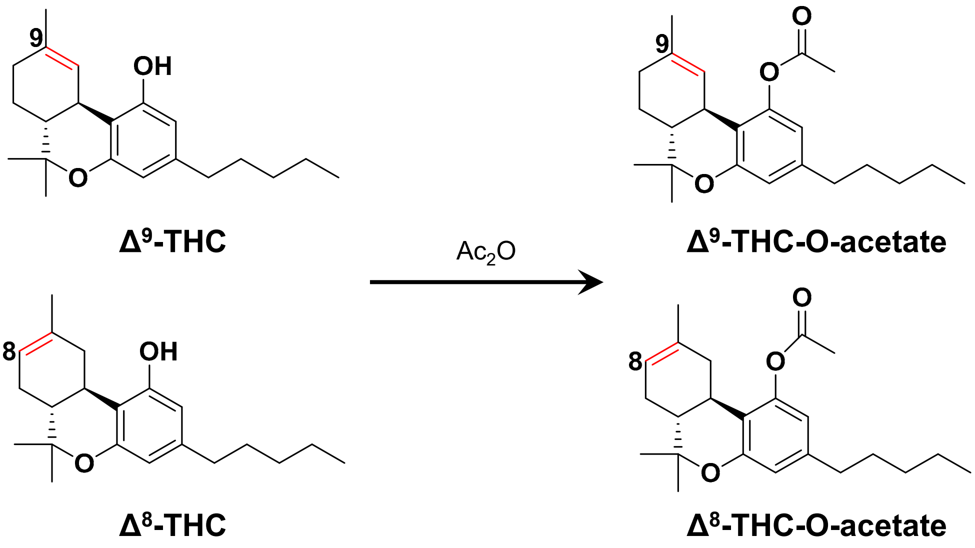

Supplementary Figure S5. Synthetic pathways of THC analogs: 9(S)/9(R)-HHC-O-acetate.

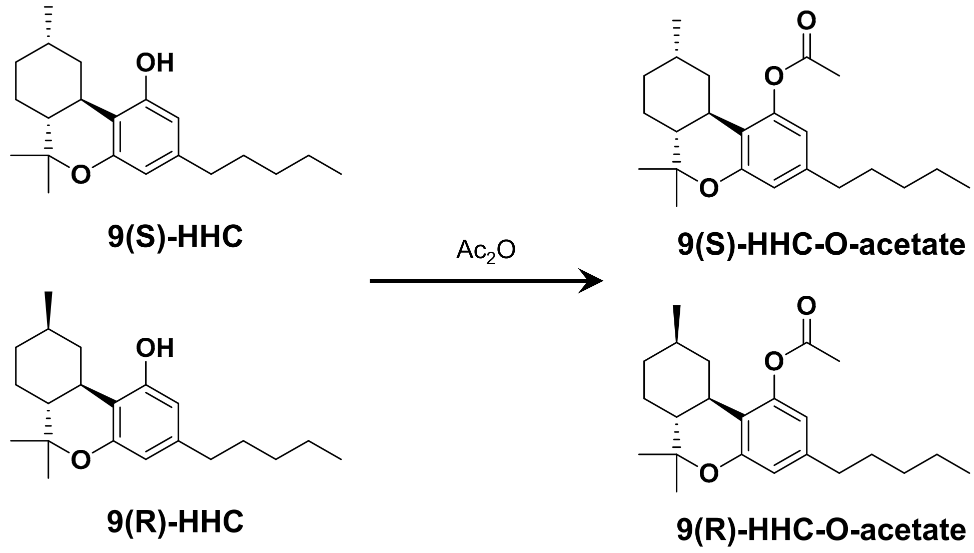

Supplementary Figure S6. LC separation of the eighteen cannabinoids using an Agilent Poroshell 120 EC-C18 column (150 mm × 2.1 mm, 2.7 µm): (A) LC-UV chromatogram at 208 nm; (B) Corresponding LC-ESI/TOFMS EICs of the eighteen cannabinoids in **Supplementary Figure S6A** using their $[M+H]^+$ ions with ±20 ppm.. The A solvent was 0.02% (v/v) formic acid. The B solvent was acetonitrile. The mobile phase contained 75.0% (v/v) B. The flow rate was 0.3 mL/min. The eighteen cannabinoids were at 1 µg/mL individual concentration.

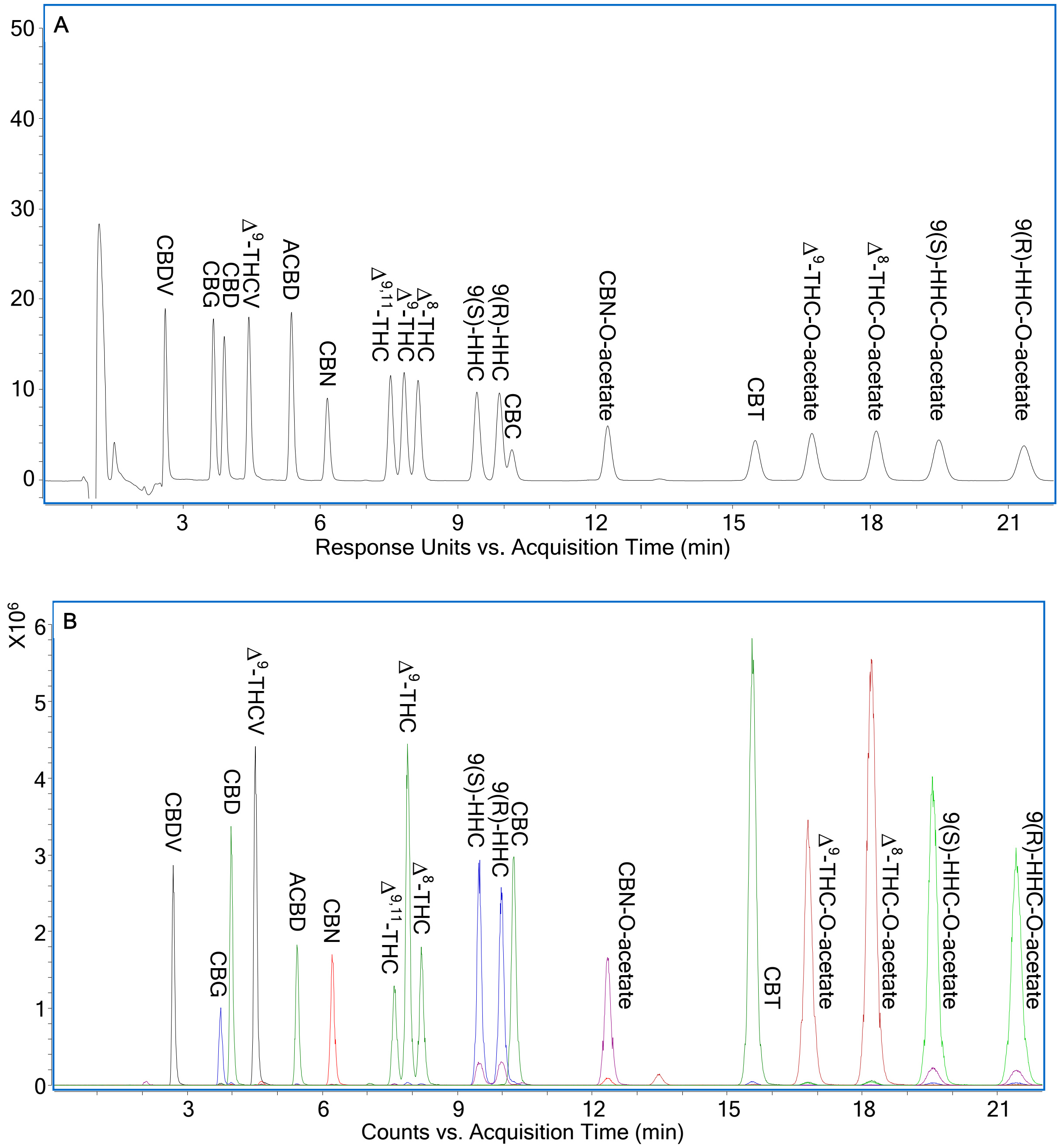

Supplementary Figure S7. UV absorption spectra of the eighteen cannabinoids that were extracted from **Figure 2A**.

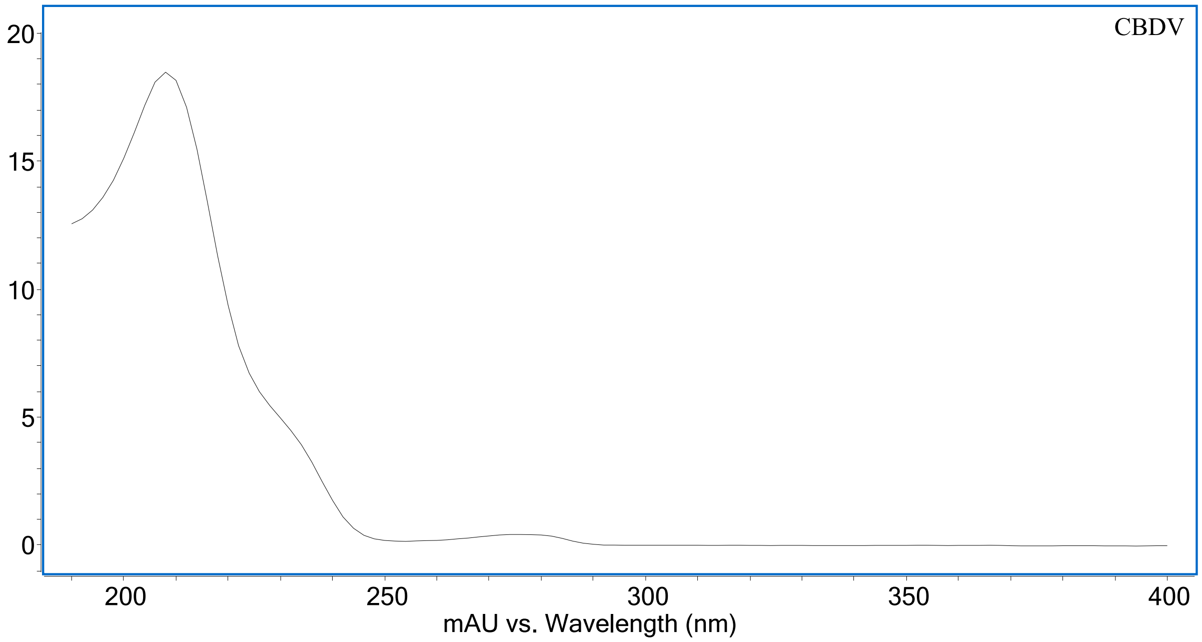

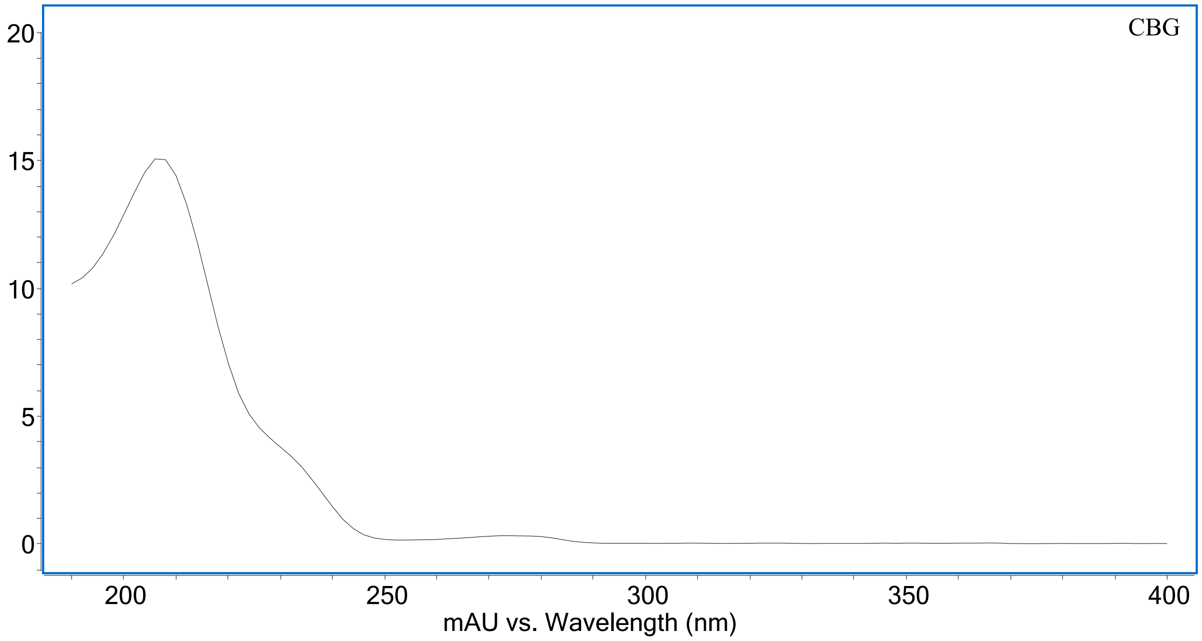

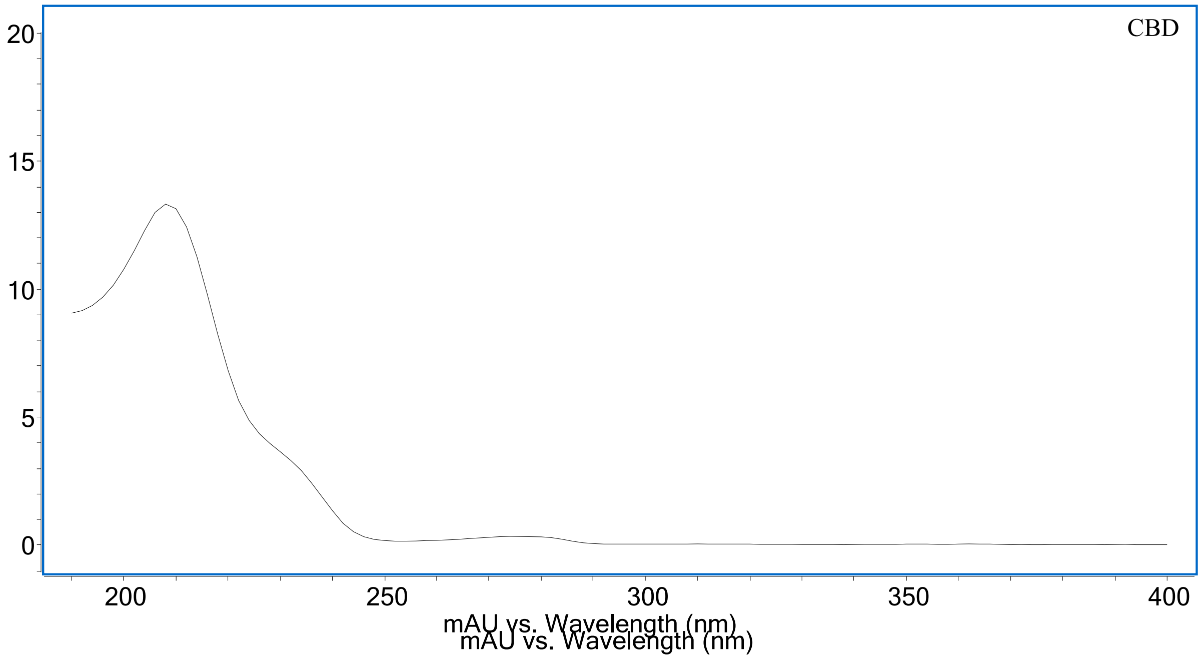
CBD
20
15
10
5
0
200
250
300
350
400
mAU vs. Wavelength (nm)
mAU vs. Wavelength (nm)

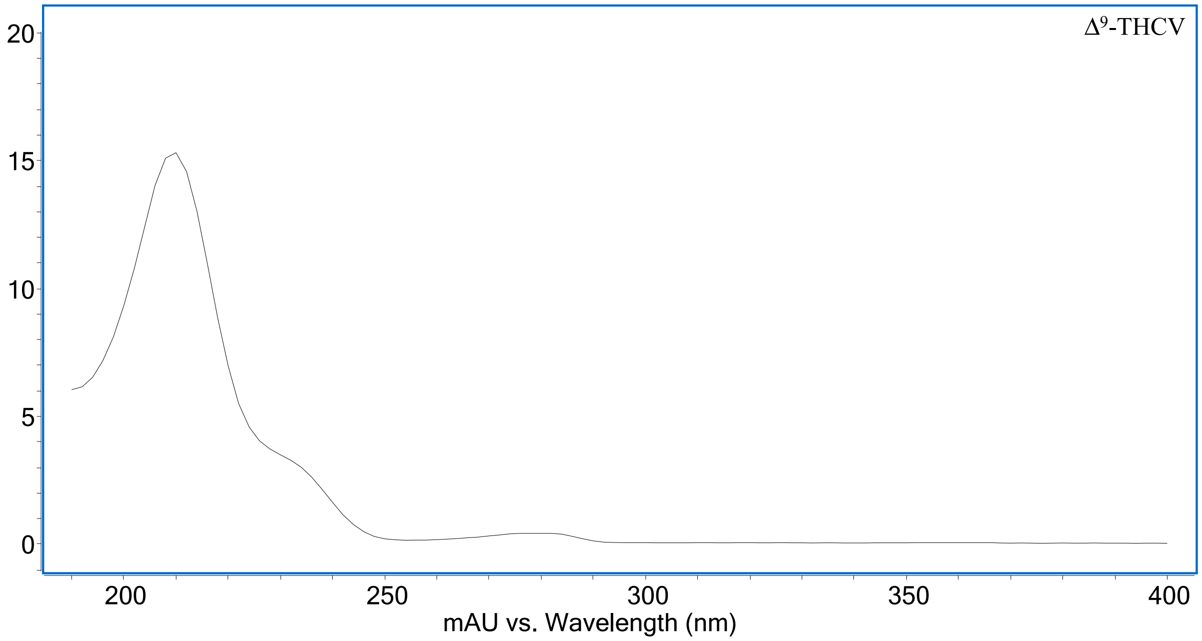
Δ9-THCV
20
15
10
5
0
200
250
300
350
400
mAU vs. Wavelength (nm)

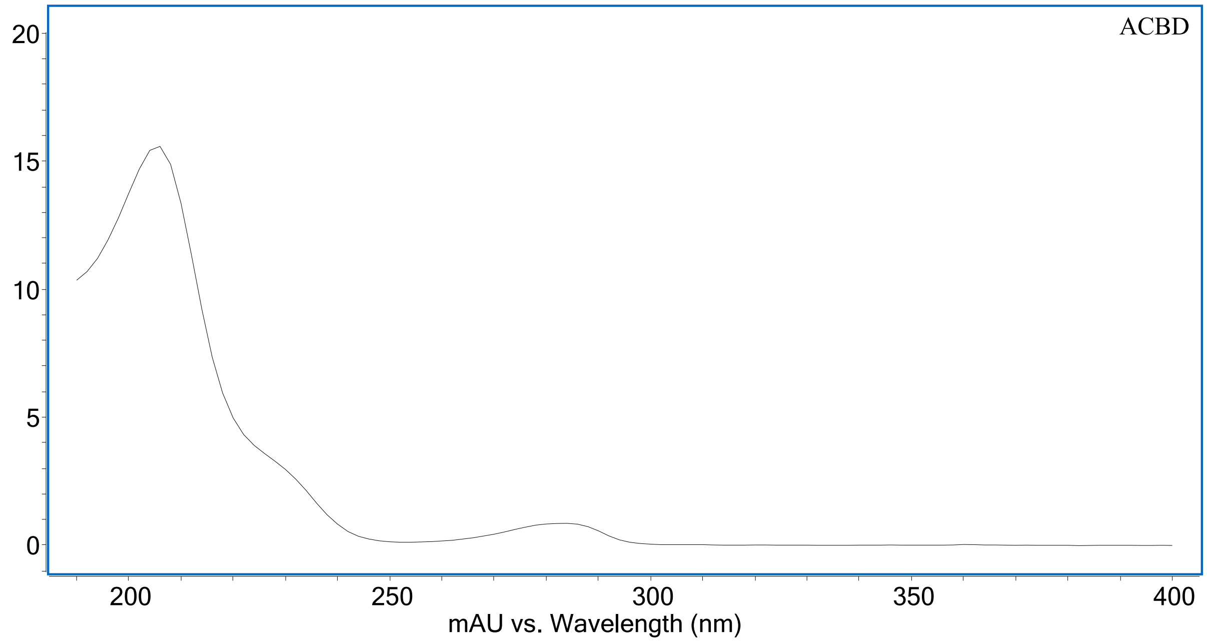
ACBD
20
15
10
5
0
200
250
300
350
400
mAU vs. Wavelength (nm)

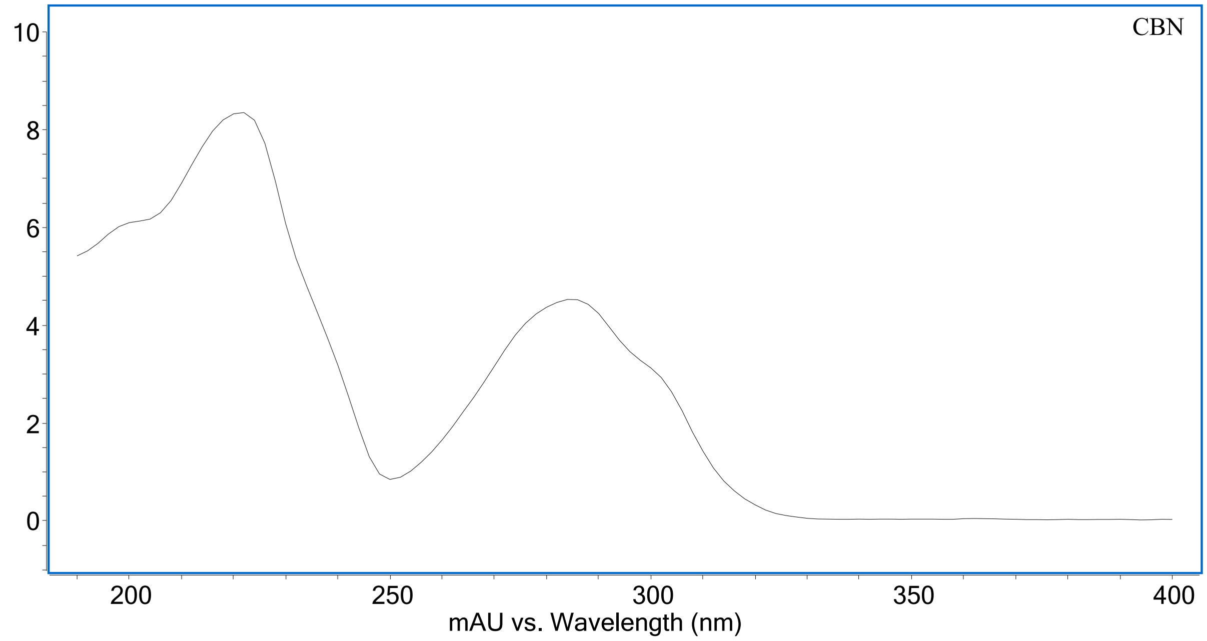
CBN
10
8
6
4
2
0
200
250
300
350
400
mAU vs. Wavelength (nm)

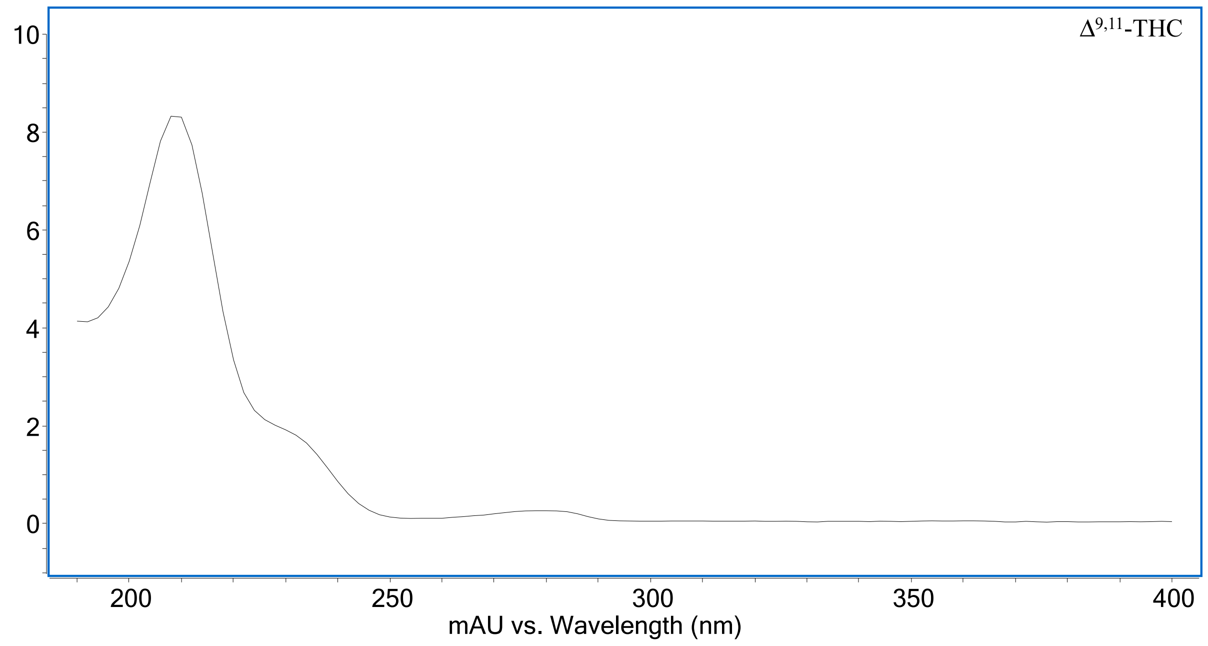
Δ9,11-THC
mAU vs. Wavelength (nm)

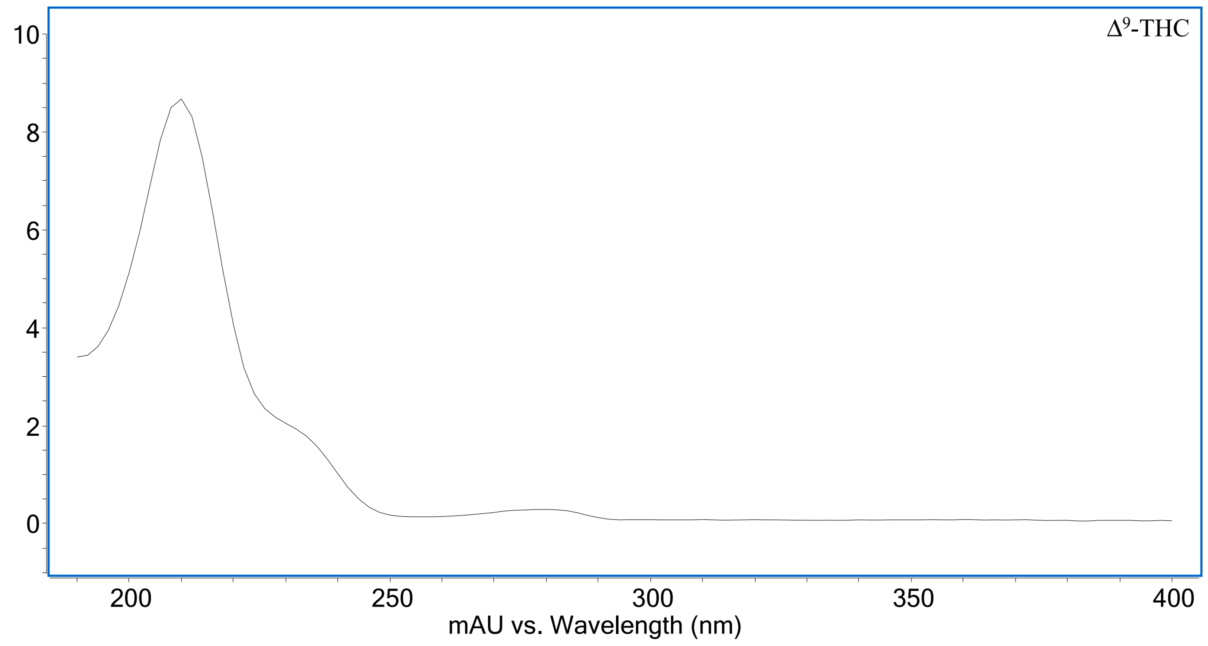
Δ9-THC
mAU vs. Wavelength (nm)

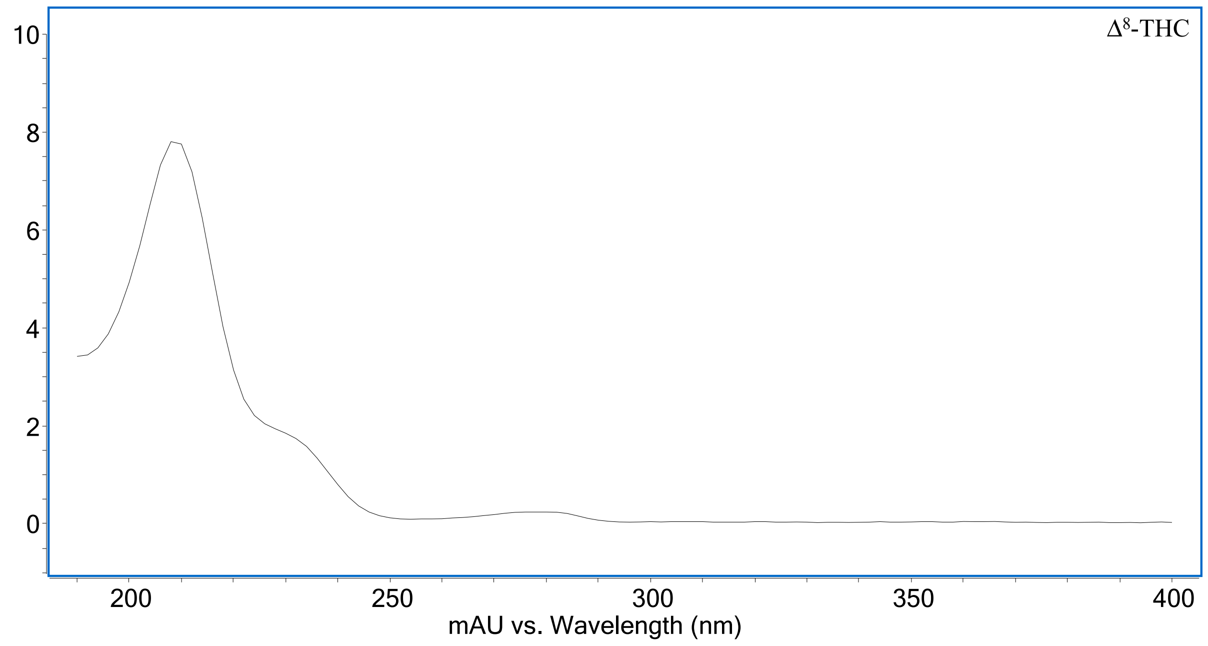
Δ8-THC
10
8
6
4
2
0
200
250
300
350
400
mAU vs. Wavelength (nm)

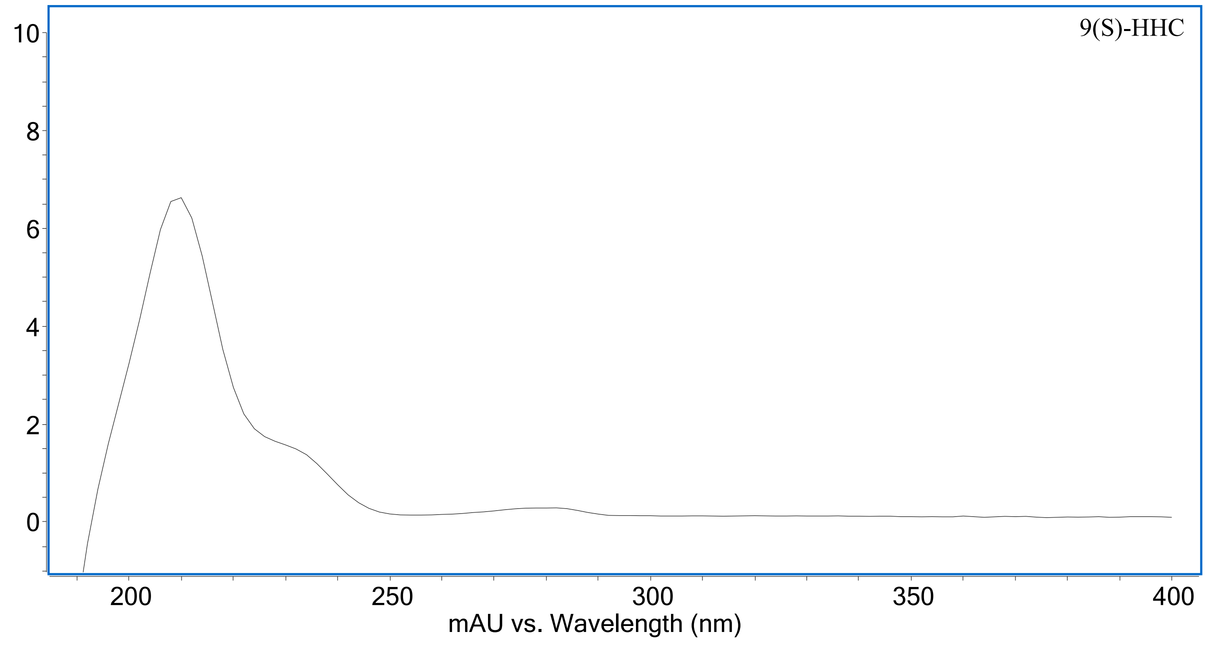
9(S)-HHC
10
8
6
4
2
0
200
250
300
350
400
mAU vs. Wavelength (nm)

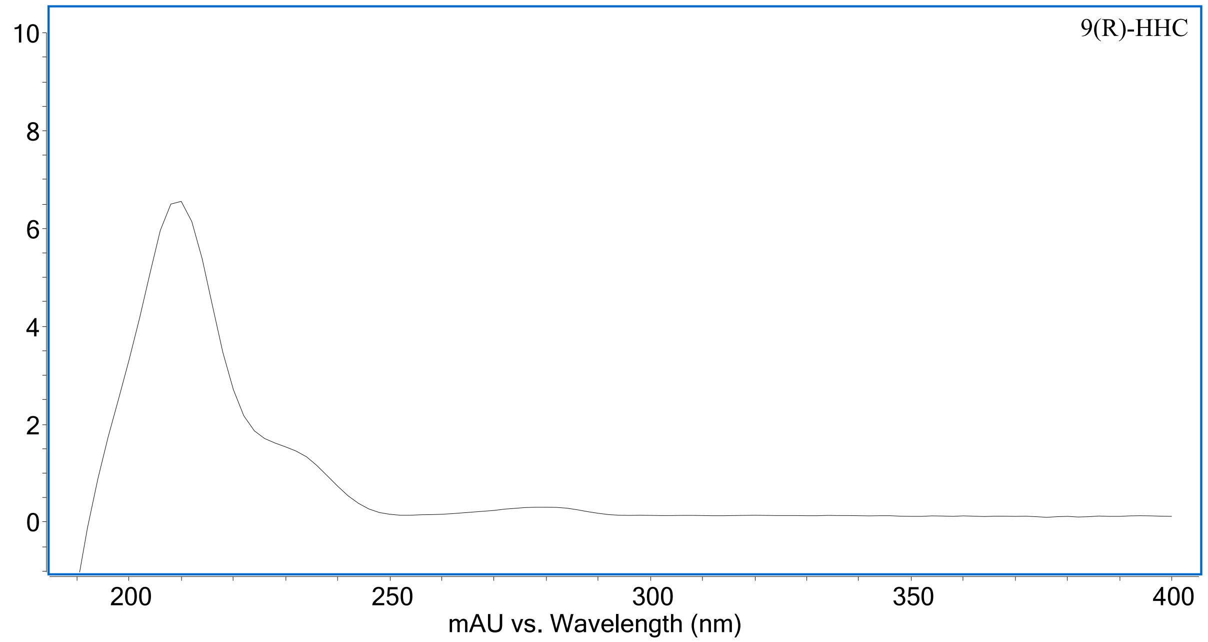
9(R)-HHC
10
8
6
4
2
0
200
250
300
350
400
mAU vs. Wavelength (nm)

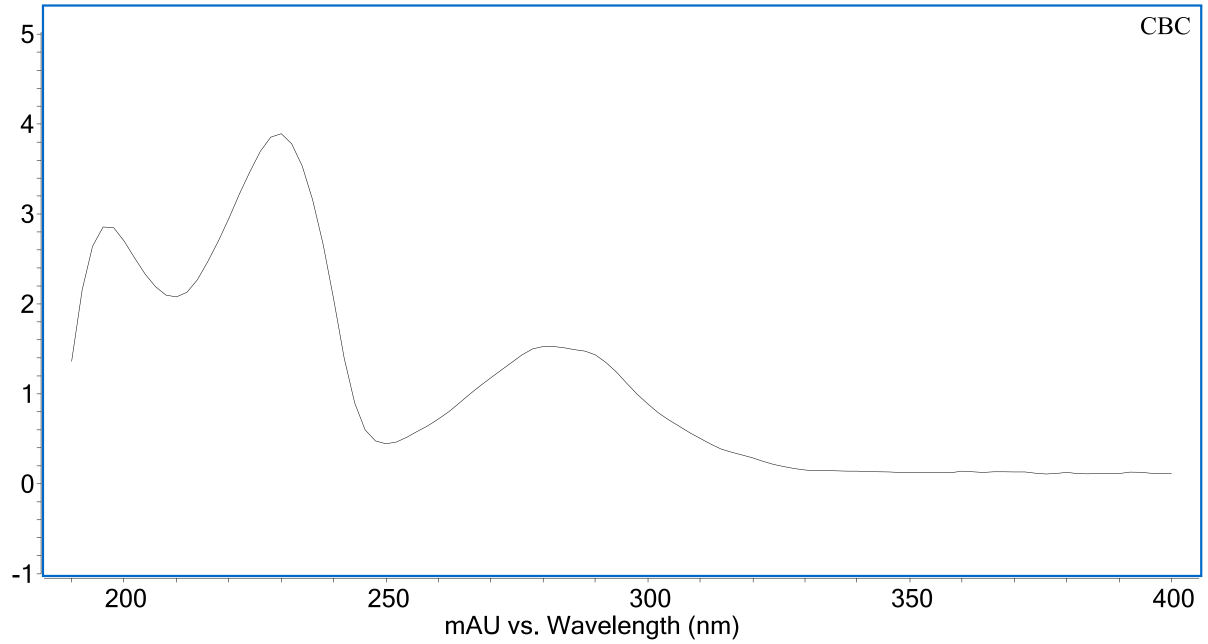
CBC
5
4
3
2
1
0
-1
200
250
300
350
400
mAU vs. Wavelength (nm)

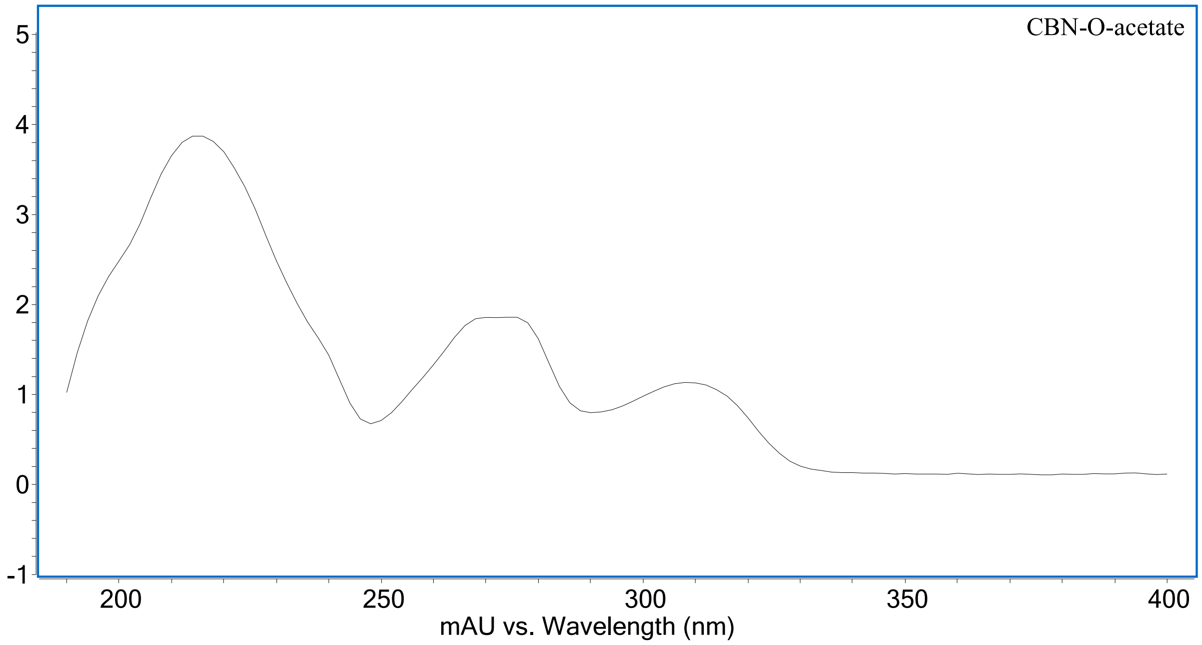

CBN-O-acetate
5
4
3
2
1
0
-1
200
250
300
350
400
mAU vs. Wavelength (nm)

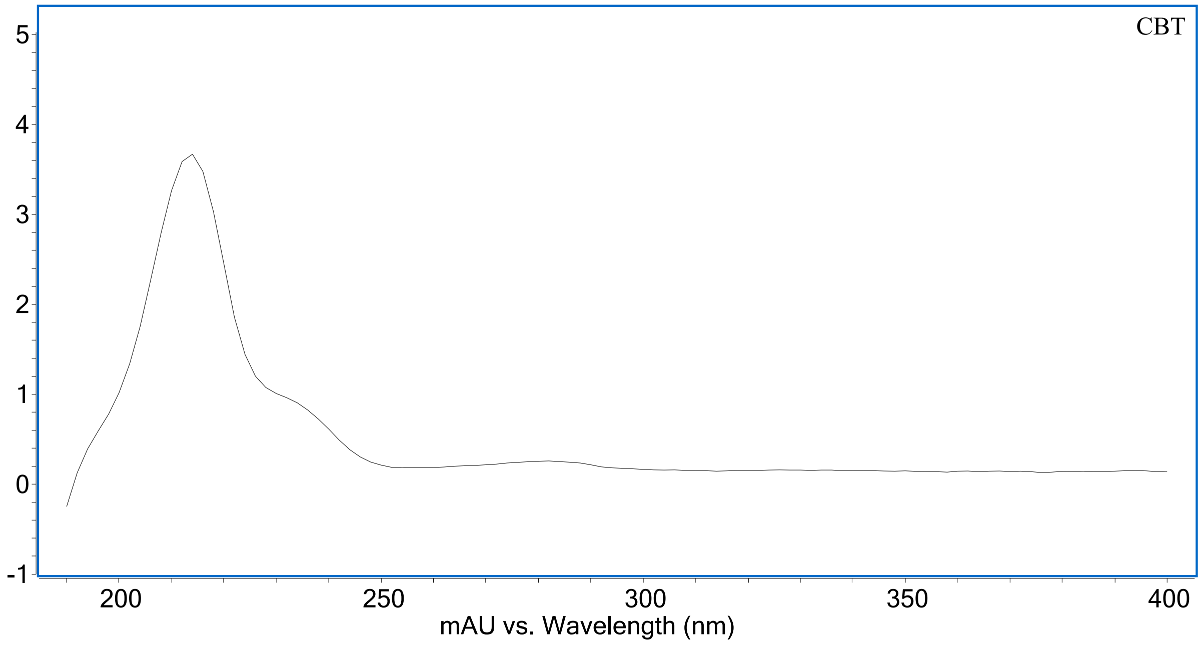

CBT
5
4
3
2
1
0
-1
200
250
300
350
400
mAU vs. Wavelength (nm)

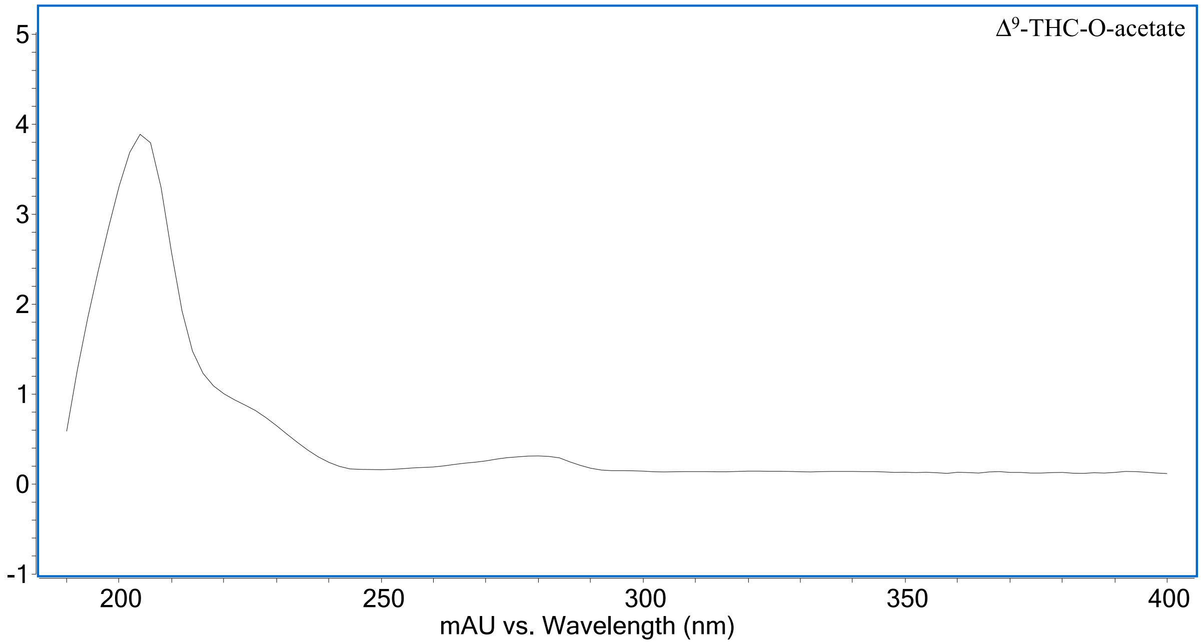
Δ9-THC-O-acetate
5
4
3
2
1
0
-1
200
250
300
350
400
mAU vs. Wavelength (nm)

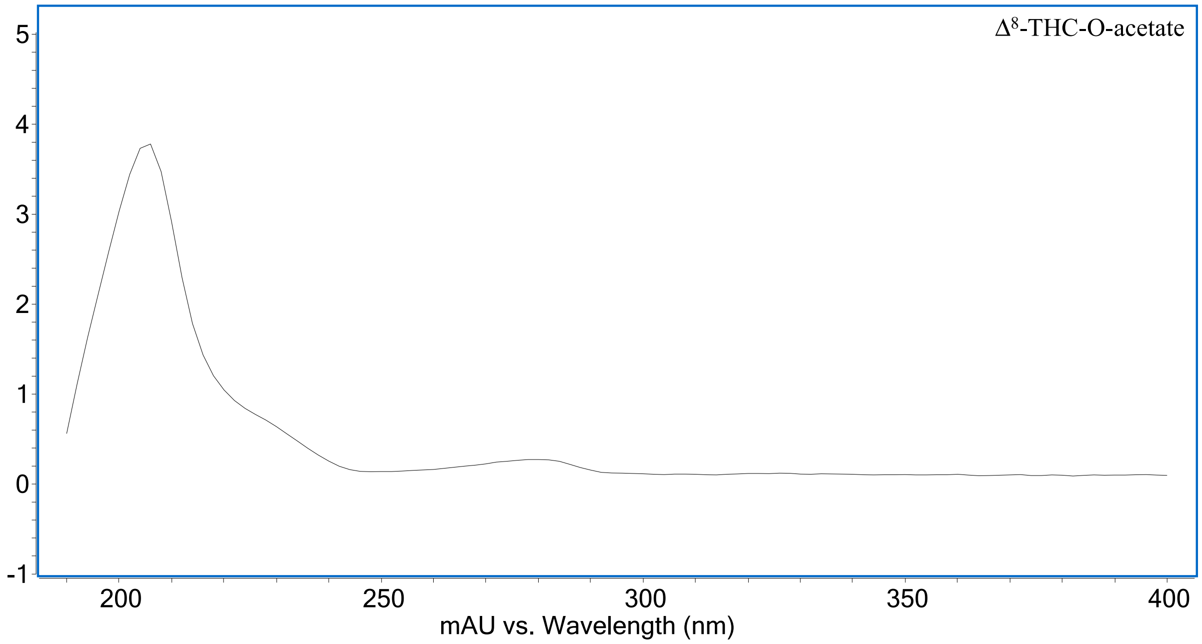
Δ8-THC-O-acetate
5
4
3
2
1
0
-1
200
250
300
350
400
mAU vs. Wavelength (nm)

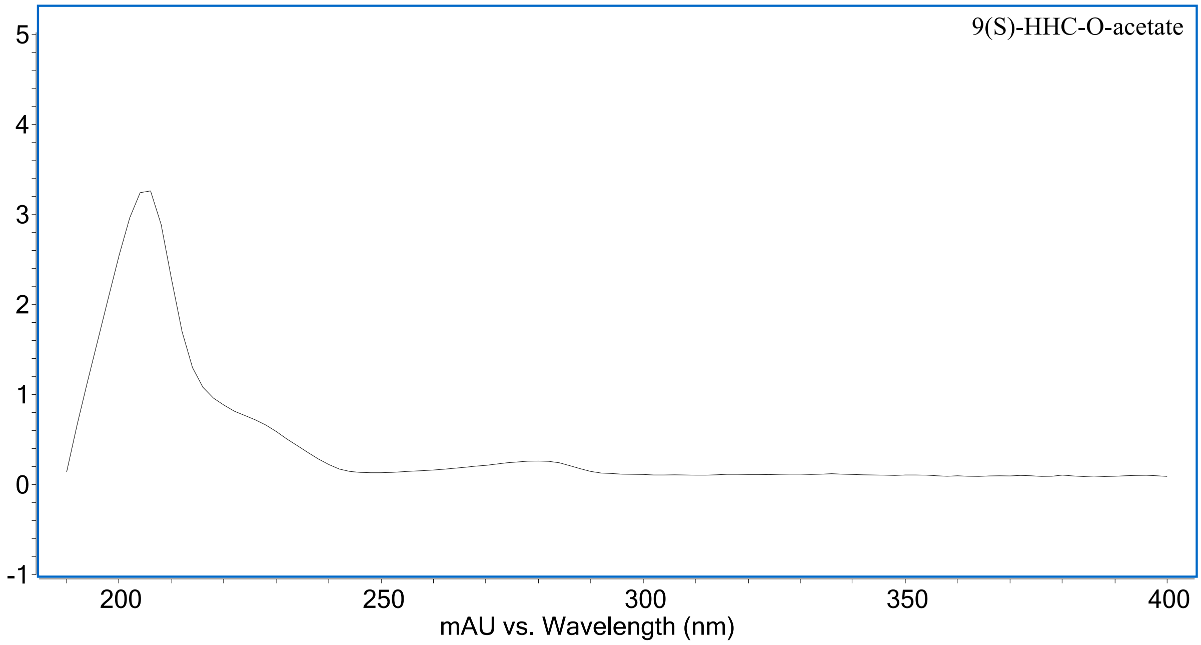
9(S)-HHC-O-acetate
5
4
3
2
1
0
-1
200
250
300
350
400
mAU vs. Wavelength (nm)

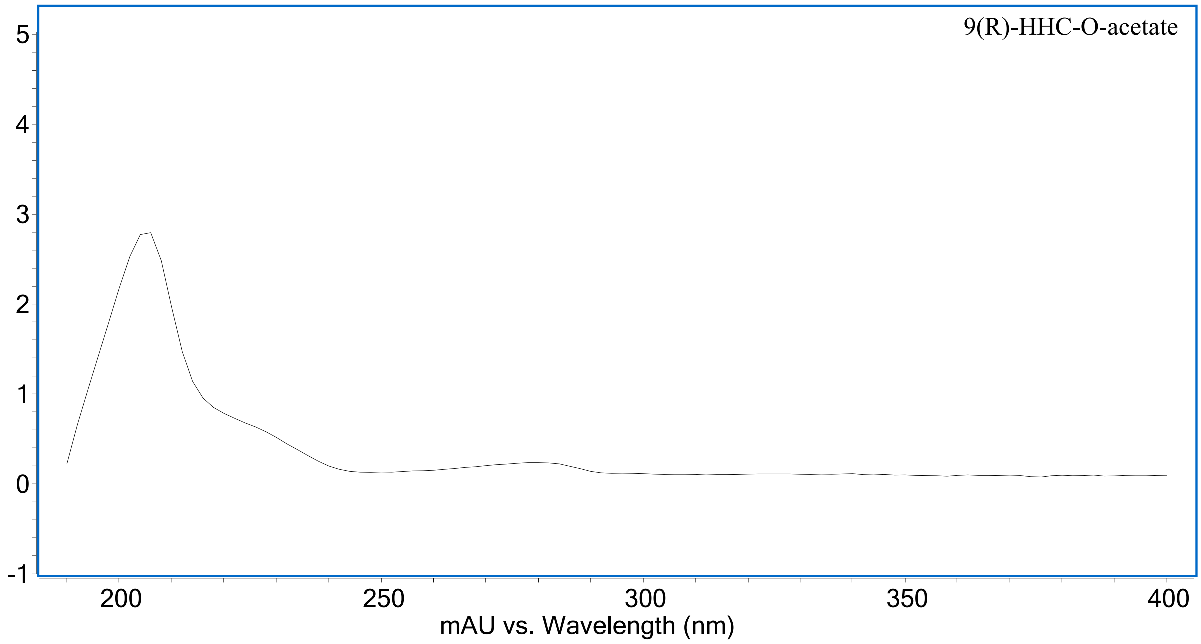
9(R)-HHC-O-acetate
5
4
3
2
1
0
-1
200
250
300
350
400
mAU vs. Wavelength (nm)

Supplementary Figure S8. ESI/TOFMS spectra of the eighteen cannabinoids that were extracted from **Figure 2A**.

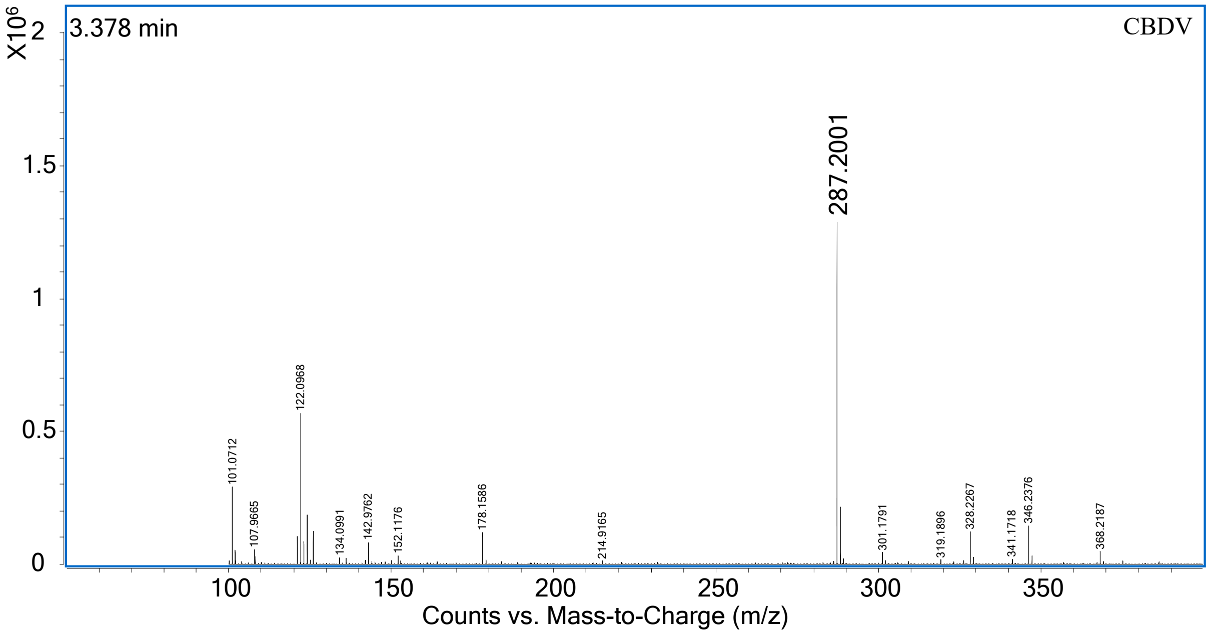

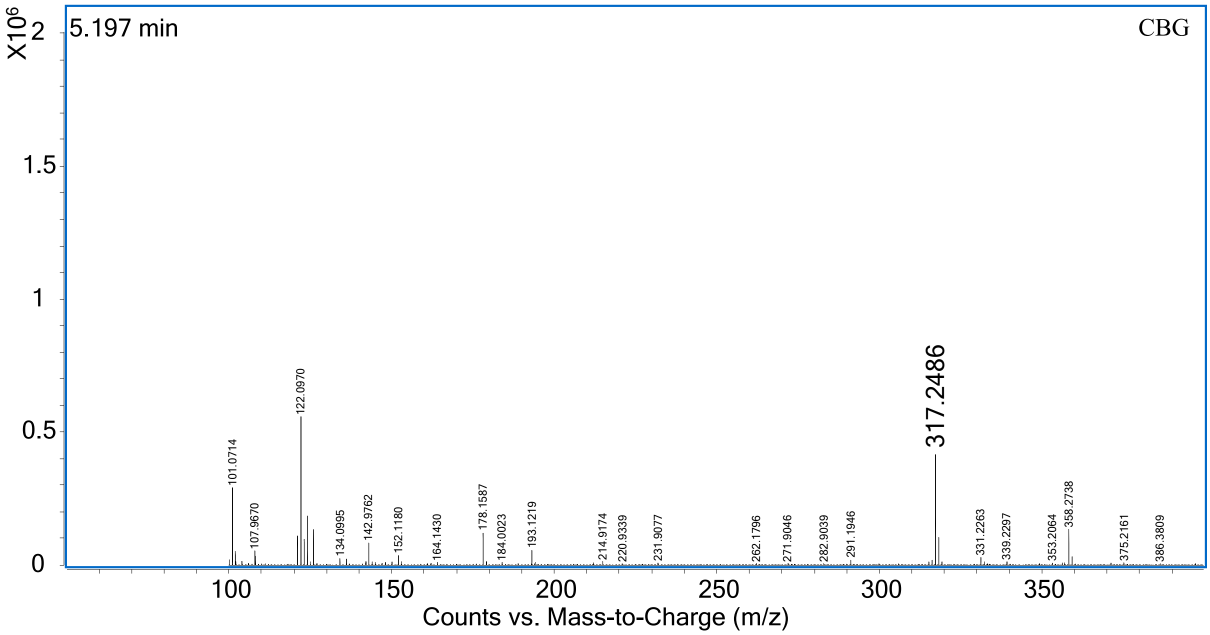

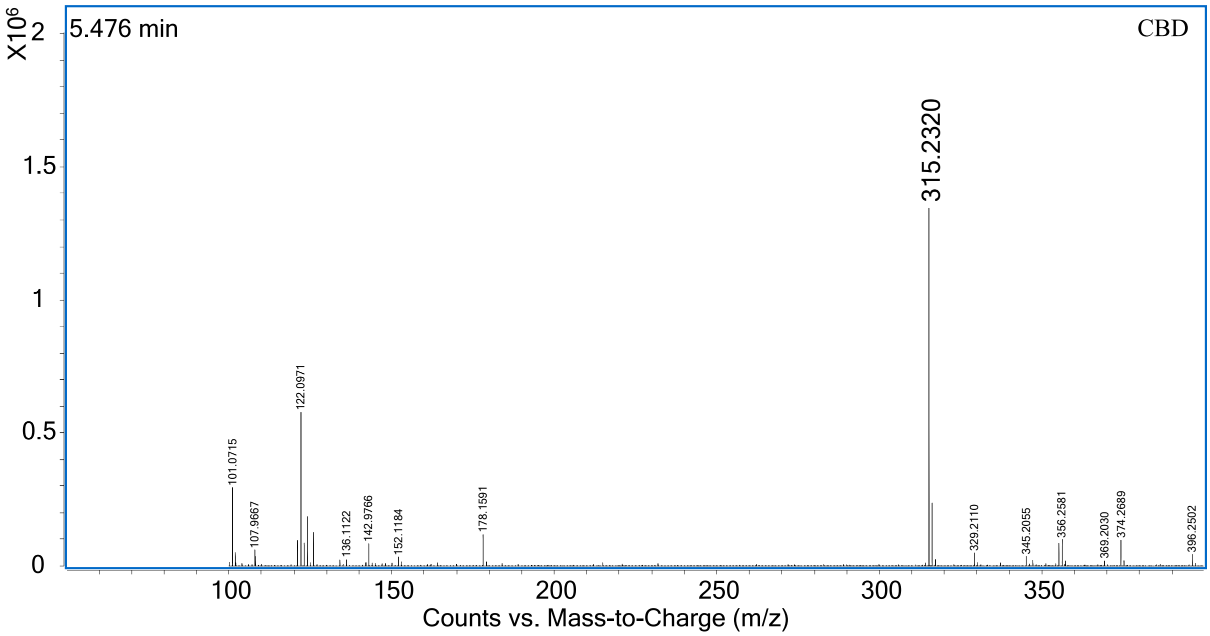
X10^6
5.476 min
CBD
315.2320
122.0971
101.0715
107.9667
136.1122
142.9766
152.1184
178.1591
329.2110
345.2055
356.2581
369.2030
374.2689
396.2502
2
1.5
1
0.5
0
100
150
200
250
300
350
Counts vs. Mass-to-Charge (m/z)

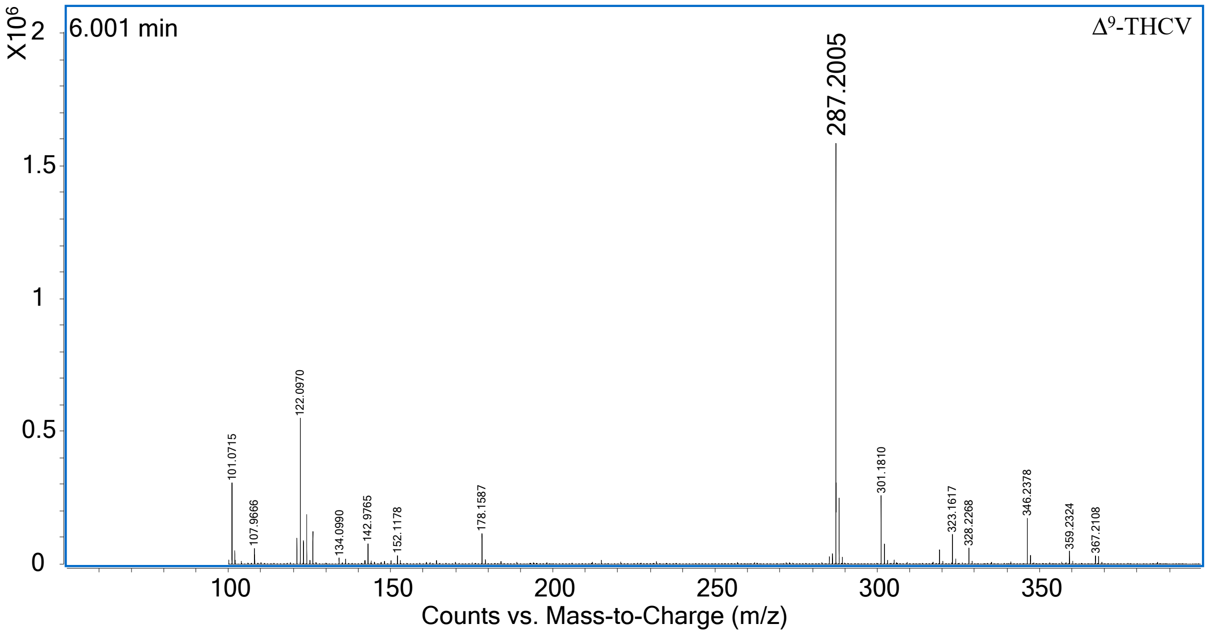
X10^6
6.001 min
Δ9-THCV
287.2005
122.0970
101.0715
107.9666
134.0990
142.9765
152.1178
178.1587
301.1810
323.1617
328.2268
346.2378
359.2324
367.2108
2
1.5
1
0.5
0
100
150
200
250
300
350
Counts vs. Mass-to-Charge (m/z)

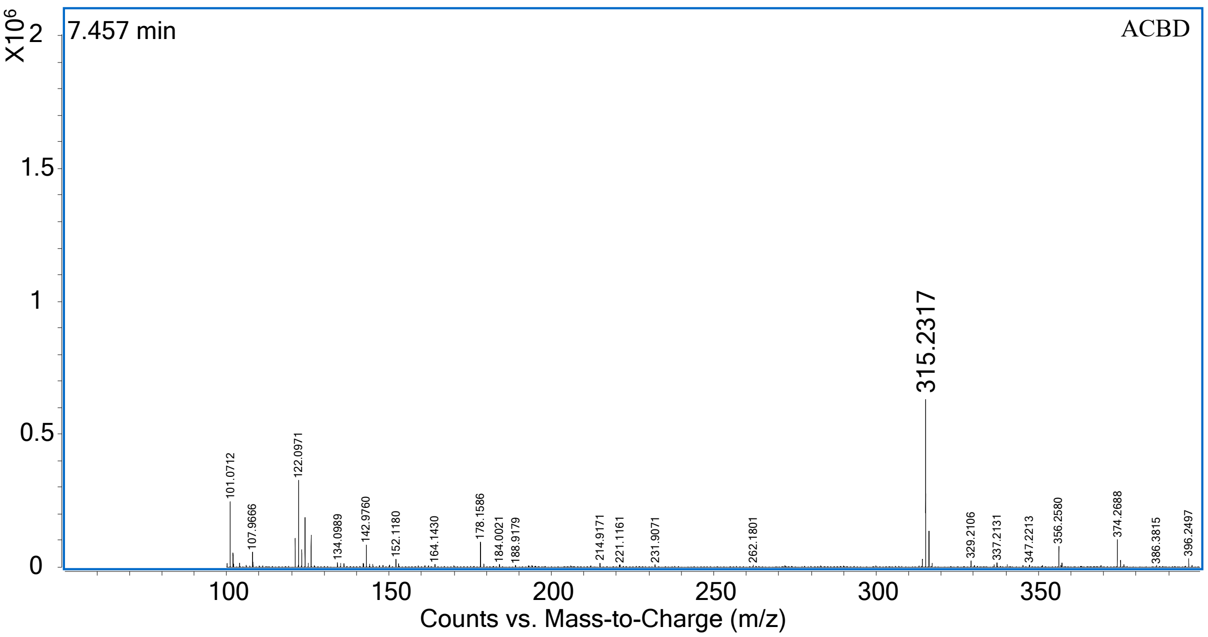
X10^6
7.457 min
ACBD
315.2317
101.0712
107.9666
122.0971
134.0989
142.9760
152.1180
164.1430
178.1586
184.0021
188.9179
214.9171
221.1161
231.9071
262.1801
329.2106
337.2131
347.2213
356.2580
374.2688
386.3815
396.2497
Counts vs. Mass-to-Charge (m/z)

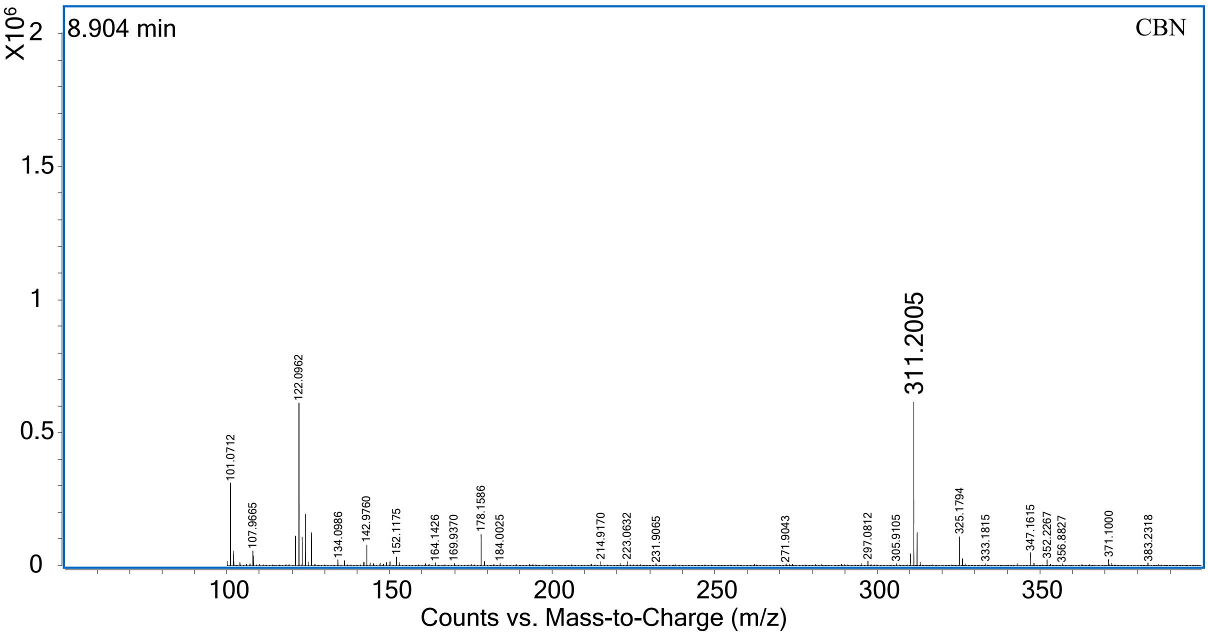
X10^6
8.904 min
CBN
311.2005
101.0712
107.9665
122.0962
134.0986
142.9760
152.1175
164.1426
169.9370
178.1586
184.0025
214.9170
223.0632
231.9065
271.9043
297.0812
305.9105
325.1794
333.1815
347.1615
352.2267
356.8827
371.1000
383.2318
Counts vs. Mass-to-Charge (m/z)

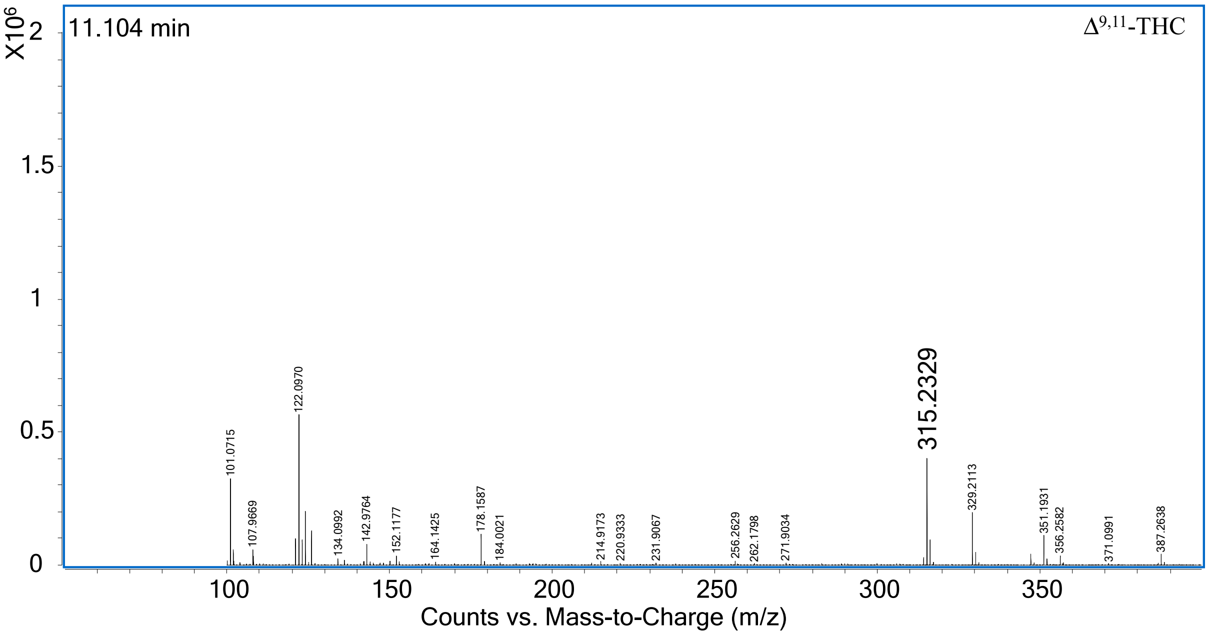
11.104 min
Δ9,11-THC
X10^6
Counts vs. Mass-to-Charge (m/z)
315.2329
122.0970
101.0715
329.2113
178.1587
351.1931
387.2638

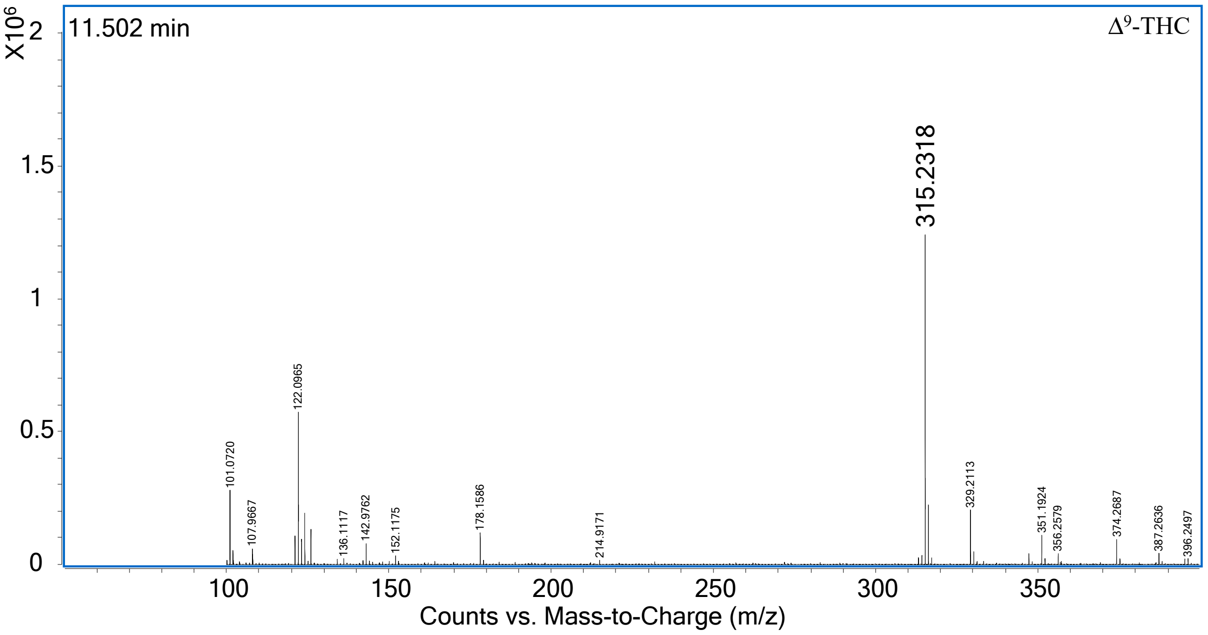
11.502 min
Δ9-THC
X10^6
Counts vs. Mass-to-Charge (m/z)
315.2318
122.0965
101.0720
329.2113
178.1586
351.1924
374.2687

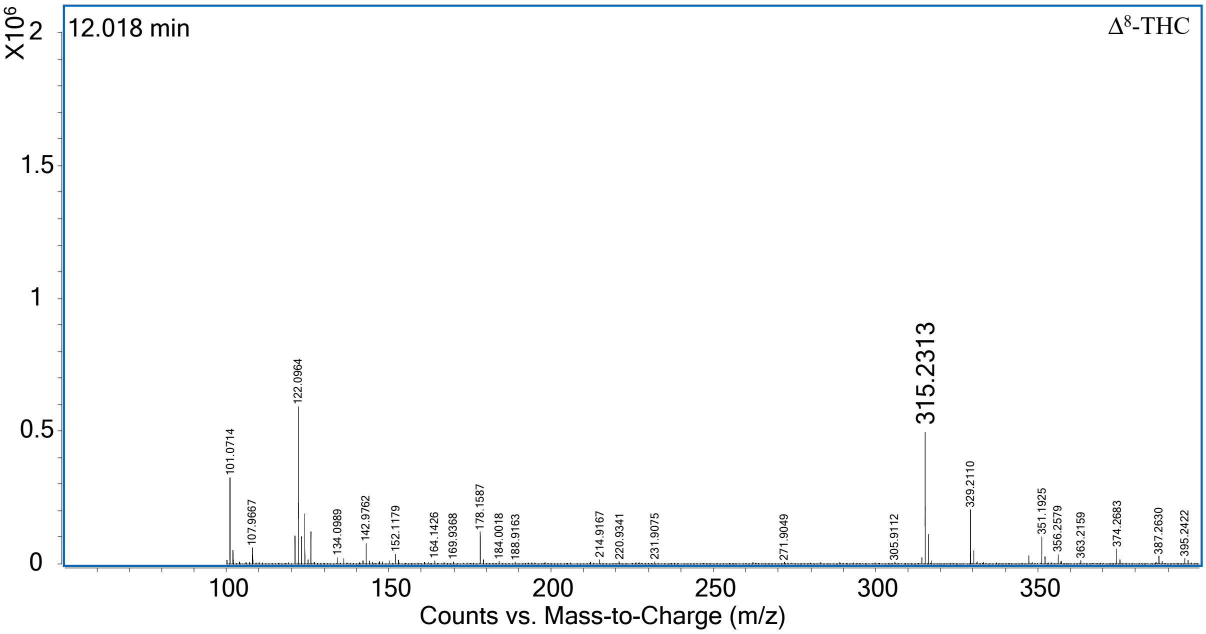
X10^6
12.018 min
Δ8-THC
315.2313
122.0964
101.0714
107.9667
134.0989
142.9762
152.1179
164.1426
169.9368
178.1587
184.0018
188.9163
214.9167
220.9341
231.9075
271.9049
305.9112
329.2110
351.1925
356.2579
363.2159
374.2683
387.2630
395.2422
Counts vs. Mass-to-Charge (m/z)

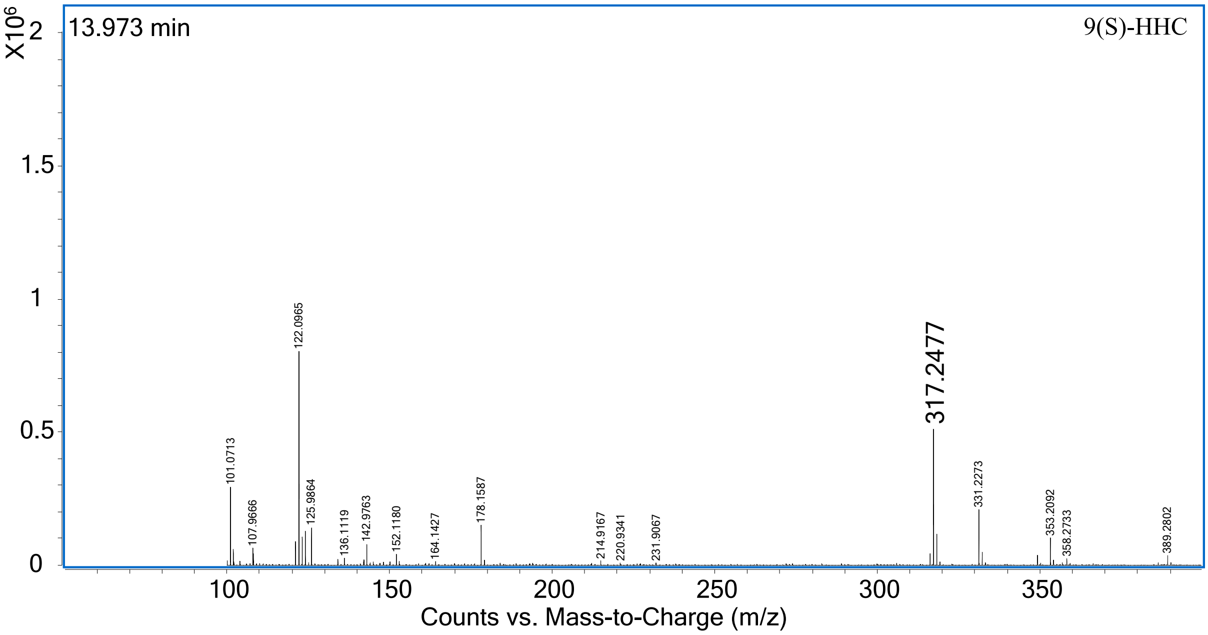
X10^6
13.973 min
9(S)-HHC
317.2477
122.0965
101.0713
107.9666
125.9864
136.1119
142.9763
152.1180
164.1427
178.1587
214.9167
220.9341
231.9067
331.2273
353.2092
358.2733
389.2802
Counts vs. Mass-to-Charge (m/z)

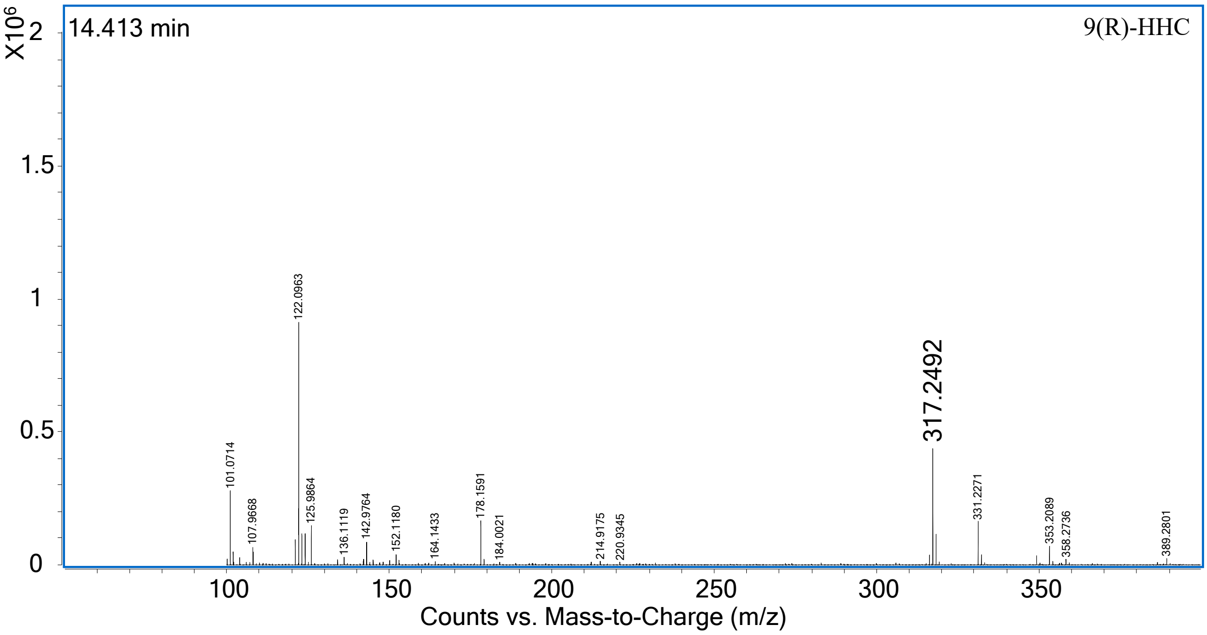
X10^6
14.413 min
9(R)-HHC
2
1.5
1
0.5
0
101.0714
107.9668
122.0963
125.9864
136.1119
142.9764
152.1180
164.1433
178.1591
184.0021
214.9175
220.9345
317.2492
331.2271
353.2089
358.2736
389.2801
100
150
200
250
300
350
Counts vs. Mass-to-Charge (m/z)

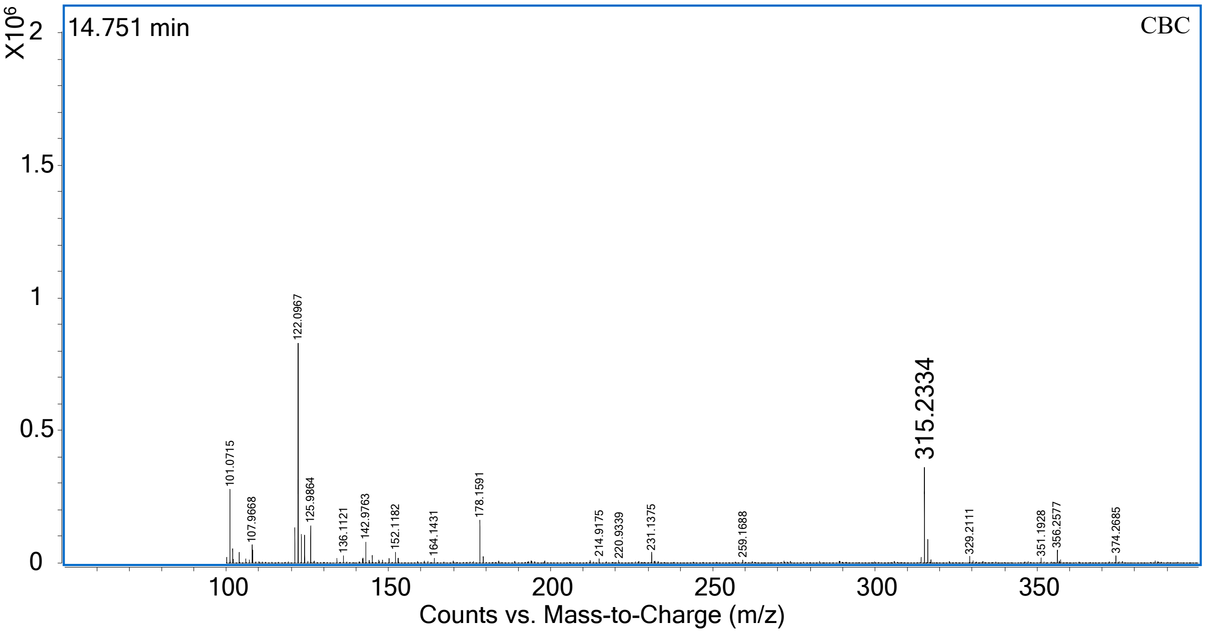
X10^6
14.751 min
CBC
2
1.5
1
0.5
0
101.0715
107.9668
122.0967
125.9864
136.1121
142.9763
152.1182
164.1431
178.1591
214.9175
220.9339
231.1375
259.1688
315.2334
329.2111
351.1928
356.2577
374.2685
100
150
200
250
300
350
Counts vs. Mass-to-Charge (m/z)

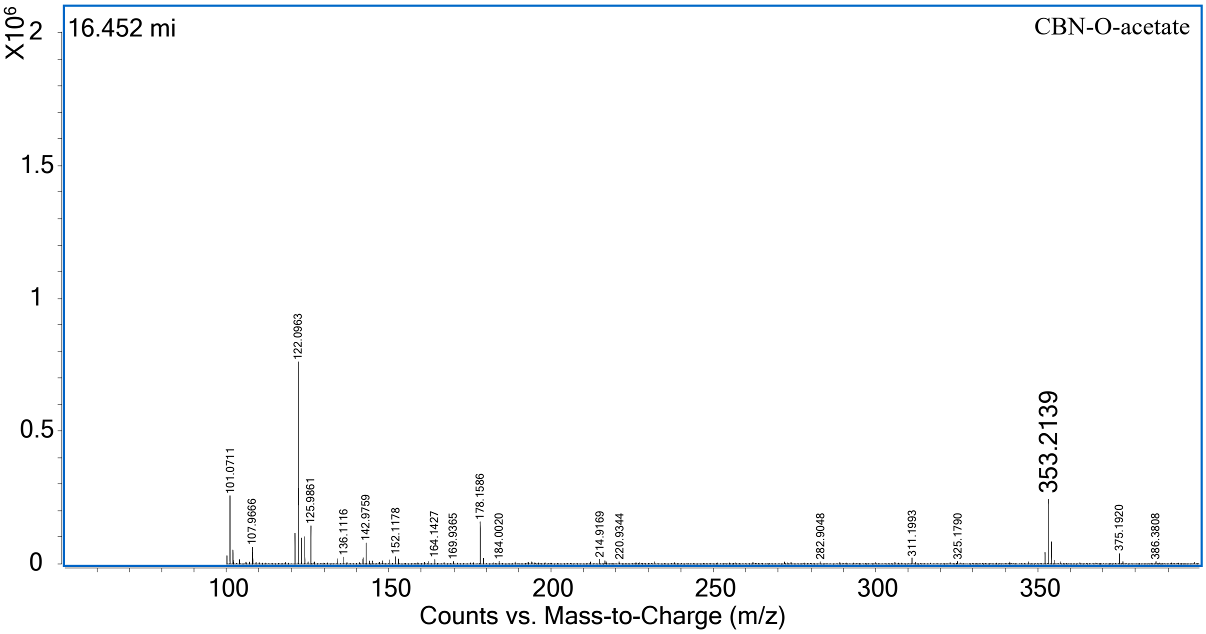
X10^6
16.452 mi
CBN-O-acetate
2
1.5
1
0.5
0
101.0711
107.9666
122.0963
125.9861
136.1116
142.9759
152.1178
164.1427
169.9365
178.1586
184.0020
214.9169
220.9344
282.9048
311.1993
325.1790
353.2139
375.1920
386.3808
100
150
200
250
300
350
Counts vs. Mass-to-Charge (m/z)

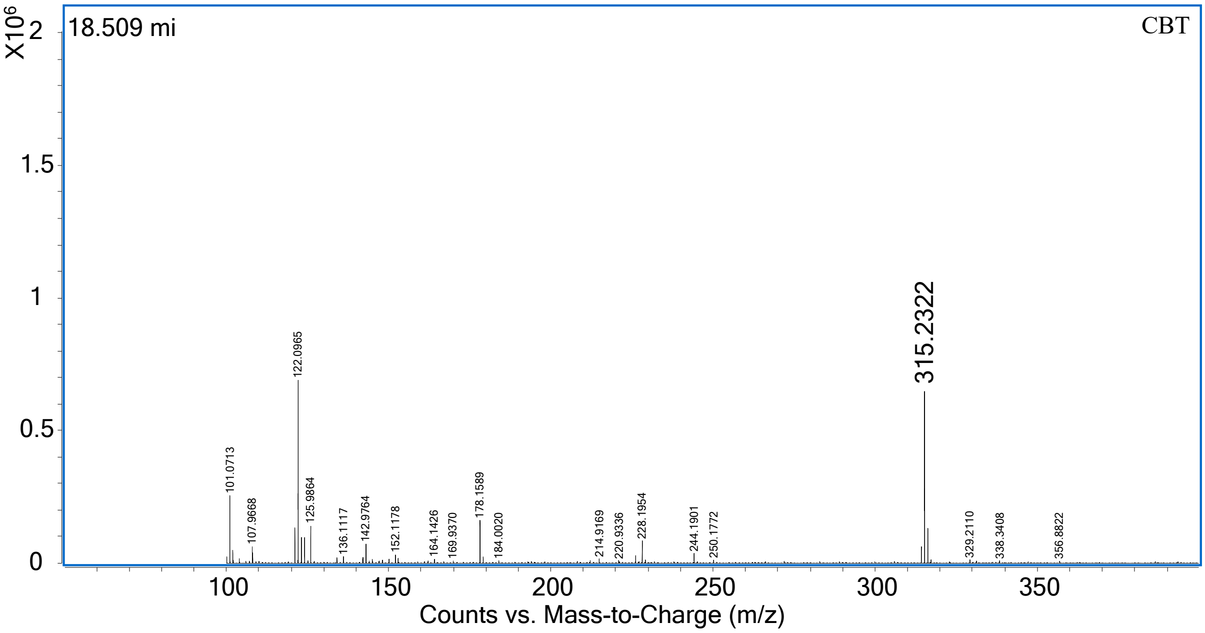
X10^6
18.509 mi
CBT
2
1.5
1
0.5
0
101.0713
107.9668
122.0965
125.9864
136.1117
142.9764
152.1178
164.1426
169.9370
178.1589
184.0020
214.9169
220.9336
228.1954
244.1901
250.1772
315.2322
329.2110
338.3408
356.8822
100
150
200
250
300
350
Counts vs. Mass-to-Charge (m/z)

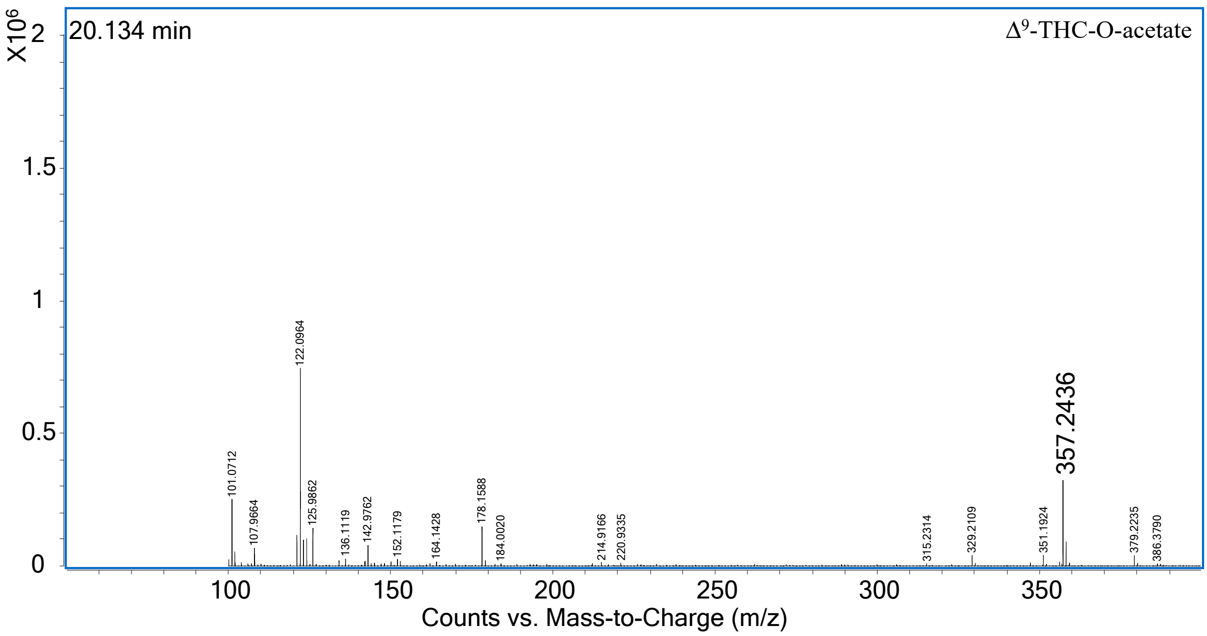

X10^6
20.134 min
Δ9-THC-O-acetate
357.2436
122.0964
101.0712
107.9664
125.9862
136.1119
142.9762
152.1179
164.1428
178.1588
184.0020
214.9166
220.9335
315.2314
329.2109
351.1924
379.2235
386.3790
Counts vs. Mass-to-Charge (m/z)

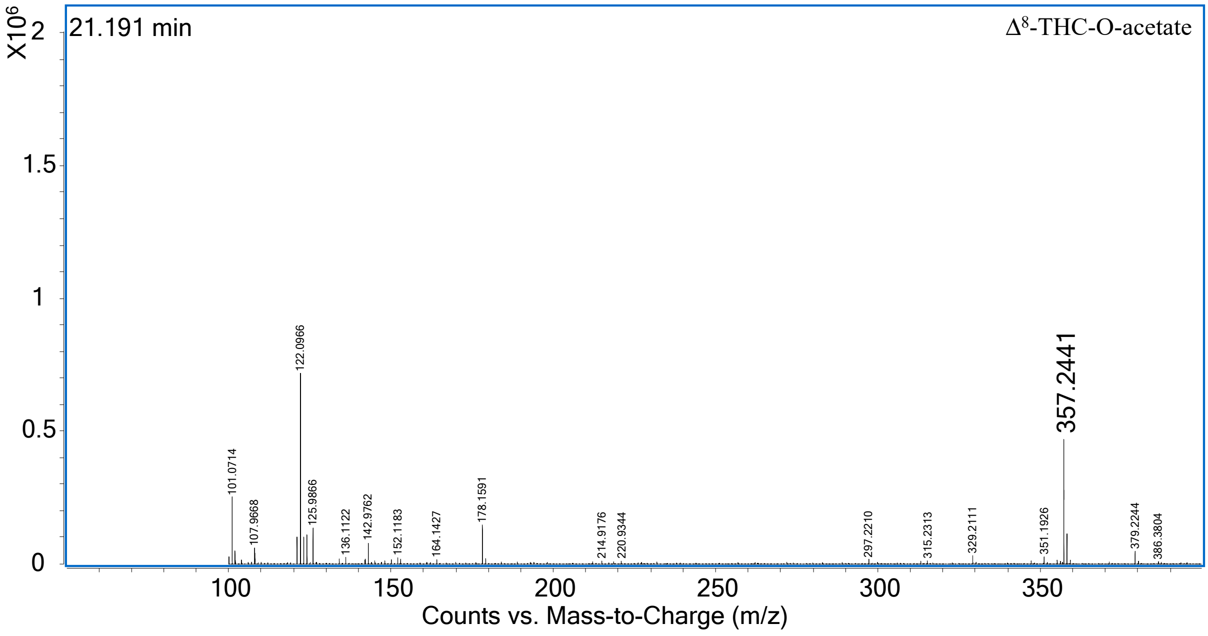

X10^6
21.191 min
Δ8-THC-O-acetate
357.2441
122.0966
101.0714
107.9668
125.9866
136.1122
142.9762
152.1183
164.1427
178.1591
214.9176
220.9344
297.2210
315.2313
329.2111
351.1926
379.2244
386.3804
Counts vs. Mass-to-Charge (m/z)

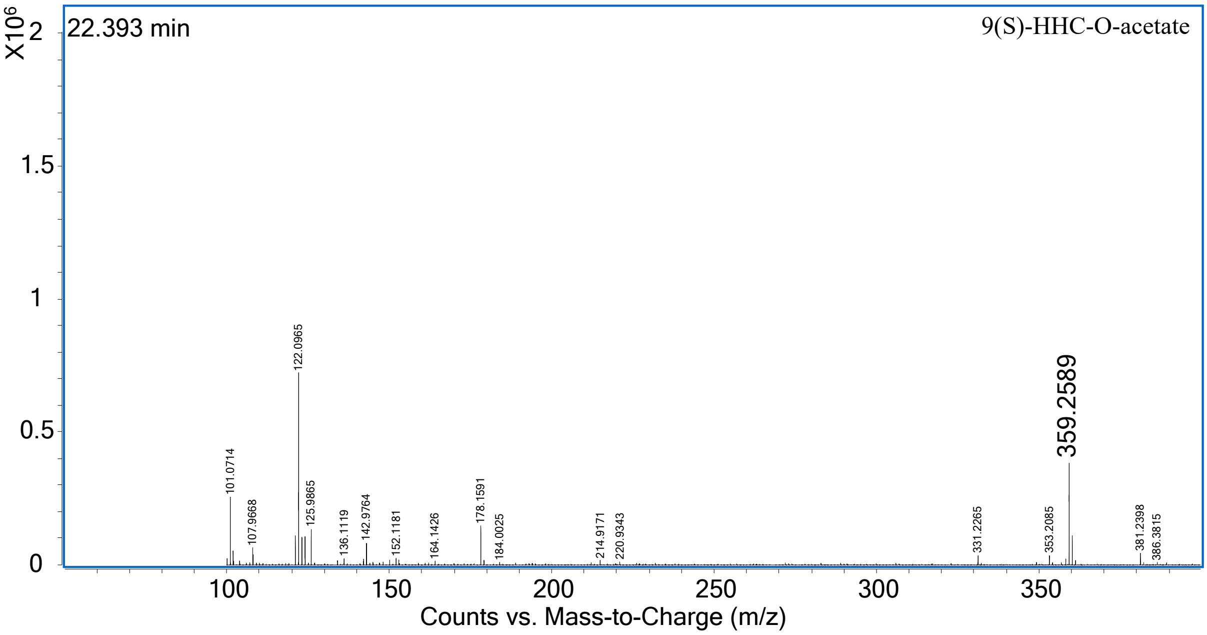

X10^6
22.393 min
9(S)-HHC-O-acetate
2
1.5
1
0.5
0
101.0714
107.9668
122.0965
125.9865
136.1119
142.9764
152.1181
164.1426
178.1591
184.0025
214.9171
220.9343
331.2265
353.2085
359.2589
381.2398
386.3815
100
150
200
250
300
350
Counts vs. Mass-to-Charge (m/z)

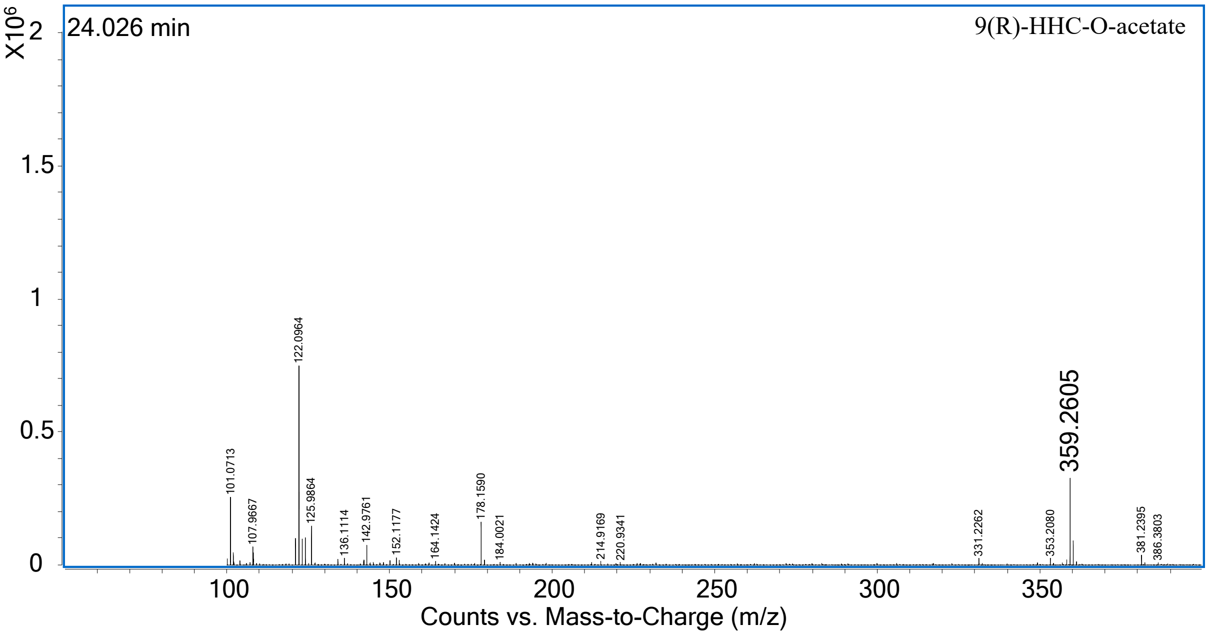

X10^6
24.026 min
9(R)-HHC-O-acetate
2
1.5
1
0.5
0
101.0713
107.9667
122.0964
125.9864
136.1114
142.9761
152.1177
164.1424
178.1590
184.0021
214.9169
220.9341
331.2262
353.2080
359.2605
381.2395
386.3803
100
150
200
250
300
350
Counts vs. Mass-to-Charge (m/z)

Supplementary Table S1. Summary of calibration curves (cannabinoid-O-acetate abbreviated as x-O-, where x = any cannabinoid).

Validation parameters		CBDV	CBG	CBD	Δ^9-THCV	CBN	$\Delta^{9,11}$-THC	Δ^9-THC	Δ^8-THC	
R^2	Day 1	0.9956	0.9949	0.9921	0.9954	0.9961	0.9947	0.9962	0.9966	
	Day 2	0.9936	0.9936	0.9927	0.9928	0.9922	0.9932	0.9957	0.9922	
	Day 3	0.9933	0.9951	0.9939	0.9922	0.9928	0.9974	0.9953	0.9945	
LLOQ	Day 1	0.1	0.1	0.1	0.1	0.1	0.1	0.1	0.1	
	Day 2	0.1	0.1	0.1	0.1	0.1	0.1	0.1	0.1	
	Day 3	0.1	0.1	0.1	0.1	0.1	0.1	0.1	0.1	
ULOQ	Day 1	50.0	50.0	50.0	50.0	50.0	50.0	50.0	50.0	
	Day 2	50.0	50.0	50.0	50.0	50.0	50.0	50.0	50.0	
	Day 3	50.0	50.0	50.0	50.0	50.0	50.0	50.0	50.0	
Validation parameters		9(S)-HHC	9(R)-HHC	CBC	CBN-O-	CBT	Δ^9-THC-O-	Δ^8-THC-O-	9(S)-HHC-O-	9(R)-HHC-O-
R^2	Day 1	0.9941	0.9968	0.9970	0.9956	0.9948	0.9952	0.9955	0.9961	0.9946
	Day 2	0.9914	0.9933	0.9949	0.9932	0.9924	0.9928	0.9934	0.9942	0.9887
	Day 3	0.9930	0.9946	0.9944	0.9928	0.9923	0.9931	0.9923	0.9941	0.9897
LLOQ	Day 1	0.1	0.1	0.1	0.1	0.1	0.1	0.1	0.1	0.1
	Day 2	0.1	0.1	0.1	0.1	0.1	0.1	0.1	0.1	0.1
	Day 3	0.1	0.1	0.1	0.1	0.1	0.1	0.1	0.1	0.1
ULOQ	Day 1	50.0	50.0	50.0	50.0	50.0	50.0	50.0	50.0	50.0
	Day 2	50.0	50.0	50.0	50.0	50.0	50.0	50.0	50.0	50.0
	Day 3	50.0	50.0	50.0	50.0	50.0	50.0	50.0	50.0	50.0

Supplementary Table S2. Accuracy (%) of the QC samples: triplicate measurements for both intraday and interday.

Conc.	Accuracy	CBDV	CBG	CBD	D^9-THCV	CBN	D^{9,11}-THC	D^9-THC	D^8-THC
0.1	Day 1	101.2	99.3	99.2	101.7	102.1	102.9	103.0	105.4
	Day 2	100.5	99.6	99.7	100.5	102.3	96.6	100.2	104.2
	Day 3	102.0	99.3	99.0	101.3	102.6	103.2	96.6	102.6
	Interday	101.2	99.4	99.3	101.2	102.3	100.9	99.9	104.1
1.0	Day 1	97.9	100.2	99.7	98.2	97.2	98.8	98.7	101.6
	Day 2	97.5	100.3	100.1	98.4	98.6	96.9	98.1	98.7
	Day 3	98.2	100.7	99.4	98.4	99.4	98.9	98.3	98.8
	Interday	97.9	100.4	99.7	98.3	98.4	98.2	98.4	99.7
50.0	Day 1	104.0	106.0	105.8	105.7	105.6	105.4	102.8	101.7
	Day 2	104.0	106.2	101.1	107.7	105.9	105.5	103.8	105.4
	Day 3	103.6	106.2	102.2	107.5	105.8	103.6	102.9	105.4
	Interday	103.9	106.1	103.1	107.0	105.8	104.8	103.2	104.2

Conc.	Accuracy	9(S)-HHC	9(R)-HHC	CBC	CBN-O-	CBT	Δ^9-THC-O-	Δ^8-THC-O-	9(S)-HHC-O-	9(R)-HHC-O-
0.1	Day 1	99.9	103.3	102.0	103.4	97.8	98.2	100.8	114.6	103.9
	Day 2	98.2	103.9	100.8	100.6	94.2	100.1	106.3	108.4	102.0
	Day 3	97.9	103.5	101.9	103.7	98.4	103.0	102.5	110.8	98.1
	Interday	98.6	103.5	101.6	102.6	96.8	100.4	103.2	111.3	101.3
1.0	Day 1	97.3	98.9	100.0	97.1	97.0	97.0	99.2	96.3	95.6
	Day 2	100.5	100.5	100.4	96.2	96.7	97.2	98.4	98.1	100.1
	Day 3	104.2	101.2	100.1	97.5	97.6	98.4	98.1	97.4	97.3
	Interday	100.7	100.2	100.2	97.0	97.1	97.5	98.6	97.3	97.7
50.0	Day 1	107.7	104.8	103.3	104.7	106.2	103.7	104.8	103.0	104.2
	Day 2	110.7	105.8	103.7	104.5	105.8	104.1	104.8	102.5	104.3
	Day 3	110.1	108.1	103.1	104.6	106.1	104.3	105.1	102.9	104.1
	Interday	109.5	106.2	103.4	104.6	106.1	104.0	104.9	102.8	104.2

Supplementary Table S3. Precision of the QC samples: triplicate measurements for both intraday and interday.

Conc.	RSD (%)	CBDV	CBG	CBD	Δ^9-THCV	CBN	$\Delta^{9,11}$-THC	Δ^9-THC	Δ^8-THC
0.1	Day 1	1.0	0.1	3.1	1.1	1.0	2.2	1.6	2.4
	Day 2	0.3	0.1	2.7	1.2	0.8	0.4	4.3	3.3
	Day 3	2.5	0.4	2.2	0.2	1.1	4.8	2.2	2.9
	Interday	0.7	0.2	0.4	0.6	0.2	3.7	3.2	1.4
1.0	Day 1	0.1	0.1	0.4	0.3	0.4	0.2	0.5	1.1
	Day 2	0.9	0.2	1.0	0.2	1.2	4.7	1.1	0.7
	Day 3	0.3	0.4	0.4	0.4	0.2	0.6	0.4	0.2
	Interday	0.4	0.3	0.3	0.1	1.1	1.2	0.3	1.7
50.0	Day 1	0.1	0.1	0.2	0.2	0.1	0.1	0.7	3.2
	Day 2	0.1	0.2	1.0	0.6	0.3	0.2	0.2	0.2
	Day 3	0.2	0.2	1.9	0.7	0.1	3.1	0.5	0.3
	Interday	0.2	0.1	2.4	1.0	0.1	1.0	0.5	2.0

Conc.	RSD (%)	9(S)-HHC	9(R)-HHC	CBC	CBN-O-	CBT	Δ^9-THC-O-	Δ^8-THC-O-	9(S)-HHC-O-	9(R)-HHC-O-
0.1	Day 1	4.1	1.3	3.0	1.8	11.5	3.1	2.4	0.8	4.2
	Day 2	3.1	4.1	1.2	3.5	0.5	4.2	1.5	4.6	11.3
	Day 3	2.3	2.4	0.5	2.5	6.8	2.4	6.5	0.5	10.6
	Interday	1.1	0.3	0.7	1.7	2.4	2.4	2.7	2.8	2.9
1.0	Day 1	1.4	0.3	0.2	0.4	0.3	0.2	2.5	0.4	0.7
	Day 2	2.4	1.0	0.9	1.2	0.6	1.6	0.6	0.7	3.1
	Day 3	2.4	0.7	2.8	0.9	0.1	2.0	2.0	1.0	1.6
	Interday	3.4	1.2	0.2	0.7	0.5	0.8	0.6	0.9	2.3
50.0	Day 1	0.3	0.1	0.1	0.1	0.3	0.7	0.1	0.1	0.1
	Day 2	1.4	0.3	1.4	0.5	0.1	0.2	0.2	0.4	0.1
	Day 3	1.9	3.7	0.7	0.4	0.2	0.5	0.3	0.4	0.5
	Interday	1.4	1.6	0.3	0.1	0.2	0.3	0.1	0.2	0.1

Supplementary Table S4. Uncertainty estimates for cannabinoid quantification.

Conc.	CBDV	CBG	CBD	Δ^9-THCV	CBN	$\Delta^{9,11}$-THC	Δ^9-THC	Δ^8-THC	
0.1	0.006	0.001	0.009	0.003	0.004	0.013	0.012	0.010	
1.0	0.02	0.01	0.02	0.01	0.03	0.10	0.03	0.04	
50.0	0.3	0.3	3.2	1.4	0.4	3.3	1.0	3.8	
Conc.	9(S)-HHC	9(R)-HHC	CBC	CBN-O-	CBT	Δ^9-THC-O-	Δ^8-THC-O-	9(S)-HHC-O-	9(R)-HHC-O-
0.1	0.011	0.010	0.007	0.010	0.027	0.012	0.015	0.011	0.033
1.0	0.10	0.03	0.06	0.03	0.02	0.05	0.07	0.03	0.09
50.0	2.8	4.0	1.6	0.6	0.5	0.9	0.4	0.6	0.6

Note:
Expanded uncertainty values (U) were calculated for each analyte using both intraday and interday precision data from QC samples, as reported in Supplementary Table S3. The combined standard uncertainty (μ_c) was obtained by pooling the standard deviations from multiple intraday replicates and combining them with the interday standard deviation using the formula:

$$\mu_c = \sqrt{SD_{intra}^2 + SD_{inter}^2}$$

Expanded uncertainty was then calculated as:

$$U = k \times \mu_c$$

with a coverage factor k=2, corresponding to an approximate 95% confidence interval. These values provide an estimate of method variability due to repeatability and intermediate precision, in line with ISO 17025 guidelines. Other sources of uncertainty such as calibration or extraction recovery were not included in this estimate but may be addressed in future full validation efforts.
